# Postoperative Pain Management in Children Undergoing Laparoscopic Appendectomy: A Scoping Review

**DOI:** 10.3390/healthcare11060870

**Published:** 2023-03-16

**Authors:** Abdalkarem Fedgash Alsharari, Farhan Faleh Alshammari, Dauda Salihu, Majed Mowanes Alruwaili

**Affiliations:** 1 College of Nursing, Jouf University, Sakaka 72388, Saudi Arabia; 2 Faculty of Nursing, University of Hail, Hail 53962, Saudi Arabia

**Keywords:** appendectomy, laparoscopy, postoperative pain, children’s

## Abstract

Laparoscopic appendectomy (LA) is one of the most commonly performed surgical procedures in children and is associated with extreme postoperative discomfort due to peritoneal inflammation and infection. The main objective of this study was to investigate the effects of postoperative pain (POP) in children after laparoscopic appendectomy. Articles describing or evaluating the control of POP in children with LA were considered eligible. All available literature such as randomized controls, prospective controls, retrospective as well as clinical studies were considered. A comprehensive search was performed in PubMed, Medline, Embase, Cochrane Library, Clinical trials.gov, and Google scholar. The initial search took place on 23 April 2021, and was updated on 24 August 2021. There were no language or date restrictions. Each of the included articles was evaluated separately by two independent reviewers. Additional papers were found by searching the reference lists of eligible studies. Eighteen papers were considered. All papers, and many of them used different methods to treat POP in children undergoing LA, such as lidocaine infusion, different analgesic approaches, ultrasound-guided transverse abdominis blockade (UGTAP), ultrasound-guided quadratus lumborum blockade (UGQLB), and comparison of open appendectomy (OA) with local anesthetics in relation to POP management in children. Laparoscopic appendectomy is the surgical procedure preferred by clinicians compared with open appendectomy in children. A multimodal analgesic approach is optimal and efficient surgical techniques such as UGBRSB, UGQLB, and UGTAP block might significantly impact POP in children except that there are contraindications. Dexmedetomidine proved to be an effective adjuvant that can enhance the effect of local anesthetics. The lack of a sufficient number of studies may be a factor affecting our confidence in the results of this study. Therefore, further evidence-based randomized control trials with a large sample size are needed to provide clarity.

## 1. Introduction

Appendicitis is common, with a lifetime risk of 6.7% to 8.6% in the United States [1], and affects people of all ages, but especially children [2]. In 2011, approximately 327,000 appendectomies were performed in U.S. hospitals, making it the 15th most expensive treatment in terms of total health care costs at USD 2441 million [3]. Appendectomy is the most commonly performed abdominal trauma surgery in children. The method LA was first defined in the 1980s [4]. Laparoscopic appendectomy has largely replaced open appendectomy in several health care facilities due to reduced hospital stays and wound infections [5]. Other “lightly invasive” procedures include needle-based surgery (port infiltration), single-incision laparoscopic surgery (SIL), and natural orifice transluminal endoscopic surgery (NOTES). Although the laparoscopic technique causes less discomfort than most other surgical methods [5], children still experience significant postoperative pain (POP) [6]. In the pediatric hospital, routine treatment for laparoscopic appendectomy includes general anesthesia, tracheal intubation, intravenous opioids, local anesthesia of the laparoscopic ports, patient-controlled analgesia (PCA), and transverse abdominal block (TAP) throughout the POP phase [7].The intensity of postoperative pain decreases between the first and third day after surgery, e.g., VAS score from 4.80 to 4.29 [8,9,10,11,12]. It has been suggested that POP may eventually leads to behavioral disturbances [13], sleep disturbances [13,14], parental frustration, and disruption of family life [15,16]. Inadequacies place a burden on the health care system and can lead to unscheduled visits to the primary care physician or clinic [17]. Inadequate POP treatment after medical intervention has been shown to be associated with chronic postoperative pain in children [18]. Chronic pain exists in 5% of children after inguinal hernia surgery and in 13.3% after other general, or orthopedic, and urologic surgeries [19,20]. Pain during laparoscopic cholecystectomy results from clinically different components [21]. These include visceral and somatic pain, usually occurring in the first 24 h [21]. In the literature, blockade of the intercostal nerves (T7-T12) along with iliohypogastric and ilioinguinal (L1) blockade has been reported to be a factor in reducing abdominal pain [21]. Given the widespread problem, there is relatively little research on the POP perceptions of children undergoing LA. Even though there is a standard of pain management, it is unclear whether or not this has resulted in a better hospital experience for children [22,23]. A plausible explanation is that the procedure is considered comparatively minor, with no tissue damage and rapid recovery. Therefore, the aim of this review was to provide an overview of the breadth of studies on POP management in children after LA.

## 2. Review Question

How is POP managed in children undergoing LA?

## 3. Inclusion Criteria

Participants: children undergoing LA.

Concept: POP control was the central theme of this study. This included a review of research that presented techniques and strategies for overcoming POP in children undergoing LA.

Intervention: Different treatment protocols or strategies for treating POP after LA in children.

Type of studies: There were no restrictions on the selection of studies, i.e., randomized control trials (RCT), clinical trials, prospective or retrospective studies were included in this review.

## 4. Method and Search Strategies

This review followed the JBI methodology for scoping reviews. A broad search was conducted on 23 April 2021, and updated on 24 August 2021, using multiple databases for material collection. PubMed, Medline, Embase, and the Cochrane Library were used to compile the report. A supplemental search was performed in Google Scholar and study registries (e.g., http://clinicaltrials.gov/ (accessed on 24 April 2021). In addition, the search strategy included all registries, and conclusions were found using the keywords “postoperative pain management in children undergoing laparoscopic appendectomy”, “children and postoperative pain management in appendectomy”, and “laparoscopic appendectomy in children” (See Supplementary File S1–S3).

### 4.1. Study Selection

During the search, all references were collected and entered into Mendeley 1.19.8, with duplicates removed, and then publications were evaluated according to the eligibility criteria. Studies that met the inclusion criteria were fully recorded and their data entered into the JBI System for the Unified Management System, Assessment, and Review of Information (JBI SUMARI; JBI, Adelaide, SA, Australia) [24]. With regard to the inclusion criterion, the full texts of the selected studies were carefully reviewed by two reviewers. In case of discrepancies, a third reviewer was consulted. After screening and study identification, the reference lists of all included studies were searched for gray literature.

### 4.2. Data Extraction

The authors designed a data extraction sheet to retrieve data from the articles used for the scoping analysis. The extraction sheet contained the following information for retrieving the articles: author, year, country of origin, purpose/aim, participants, study methodology, and postoperative pain. Two authors performed ongoing cross-checks to ensure the accuracy of the results extracted from the randomly selected records.

### 4.3. Data Analysis and Presentation

Results were documented and summarized in both narrative and tabular form, including an informative summary. In addition, the results were categorised in relation to the theme of the study (access to postoperative pain management in children after laparoscopic appendectomy). Our approach to synthesis was based on established literature from other investigators, which contributed to a realistic interpretation of the analysis results.

## 5. Results

### 5.1. Study Inclusion

**Figure 1:**
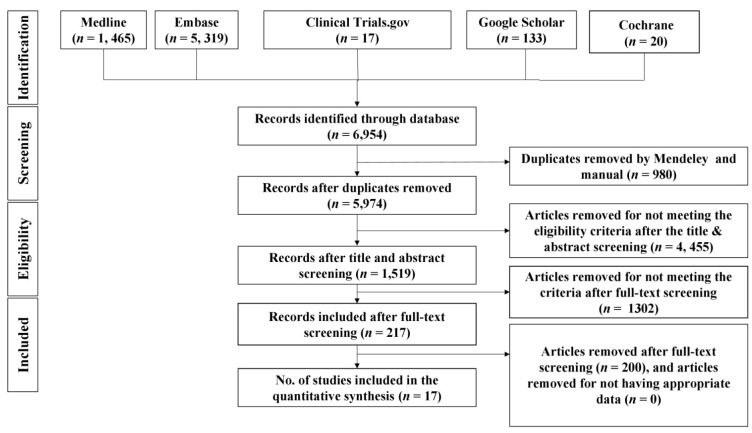
PRISMA flow diagram.

As shown in Figure 1, a total of 6954 results were generated from the five electronic databases. After deduplication with Mendeley and manual search, 5974 records were found. After screening titles and abstracts against the inclusion criteria, 1519 records were found as follows: 1302 were removed for not meeting the criteria for full-text screening; 217 of these documents were subjected to detailed full-text review by two different authors, while a third reviewer was asked to resolve any discrepancies between them. This resulted in the exclusion of 200 documents that did not meet the inclusion criteria (Supplementary Material). Seventeen articles (*n* = 17) that met the inclusion criteria were included in this review.

### 5.2. Characteristic of Included Studies

The 17 included papers were published between 1996 and 2020, and virtually all mentioned different approaches to the treatment of POP in children with LA, i.e., lidocaine infusion, different analgesic approaches, ultrasound-guided transverse abdominis block (UGTAP), ultrasound-guided quadratus lumborum block (UGQLB), and comparison of open appendectomy (OA) with LA in relation to the treatment of POP in children [7,10,22,23,24,25,26,27,28,29,30,31,32,33,34,35,36,37,38]. As shown in Table 1, a total of 1754 samples were included in the study, of which 61.86% were male. The age of the participants ranges from 2 to 19 years. The visual analog scale appears to be the most commonly used scale for pain assessment (VAS). The randomized controlled trial (*n* = 847.1%) appears to be the most commonly used design, whereas bupivacaine (0.25% and 0.5%) was the most commonly used solution for infiltration of surgical sites. Midazolam at a dose of 0.05 mg/kg was the most commonly used premedication. A wide range of analgesics was used for induction, including fentanyl (0.5 mcg/kg to 13 mcg/kg), acetaminophen 15 mg/kg, metamizole 10 mg/kg, and paracoxib 1 mg/kg. Intraoperatively, fentanyl (0.5–3 mcg/kg), morphine (0.1 mg/kg), ketorolac (0.3–0.5 mg/kg), paracetamol (15 mg/kg), paracoxib (1 mg/kg), and decocine (0.10 mg/kg) were used as analgesics. Postoperatively, children received paracetamol (15 mg/kg), nalbuphine (0.1 mg/kg), ketorolac (0.5 mg/kg), morphine (15–20 mcg/kg, and, or 0.02–0.05 mg/kg), piritramide (0.05 mg/kg), hydromorphone (4 mcg/kg), pethidine (1 mg/kg), and tramadol if needed.

### 5.3. Review Findings

#### POP Management in Children after LA

##### Effect of Commonly Used Analgesics in Children

Postoperative pain might be a major problem in children undergoing LA. In our study, multimodal analgesic treatment (MMAT) seems to be the strategy most commonly used by physicians to overcome POP after LA in children. In a three-arm study, dezocine, fentanyl, and normal saline were each administered 30 min before the end of surgery [25]. It was found that MAP (multiple activity pain) and FLACC (face, leg, activity, cry, consolability) of the dezocine groupwere significantly different compared with fentanyl and normal saline arms(*p* > 0.05). In addition, they concluded that intravenous administration of dezocine (IV) before the end of surgery can significantly reduce POP and agitation in children undergoing surgery LA.

The post-operative analgesic efficacy of ketorolac (0.5 mg/kg), diclofenac (2 mg/kg), and paracetamol (15 mg/kg), were also evaluated using a three arms study [26]. FPS-R (face pain scale-revised) was used to assess the extent of pain 30 and 60 min after surgery. A large difference (*p* > 0.05) existed between classes, with patients in the ketorolac group (77.5%) and the diclofenac sodium group (42.5%) achieving pain score 2 and acetaminophen frequency (37.5%) achieving pain score 4. Pain scores at 60 min also differed significantly between groups (*p* > 0.05), with the ketorolac group (72.5%) scoring 0, the diclofenac sodium group (62.5%) scoring 2, and paracetamol (37.5%) scoring 6 most frequently. As shown in Table 1, the experimental group apparently experienced less pain (2.19 ± 0.32) than the control group (3.56 ± 0.37) [27]. The 60-min pain scores seem to be lower in those using ketorolac, as 75% of them reported a pain score of 0, in contrast to 62.5% for paracetamol and 37.5% for diclofenac sodium with a score of 2 and 6, respectively [26]. In the recovery rooms, it was also reported that postoperative pain in the intervention group had a median pain score of 0 compared with 2 (95% CI 0–3, *p* = 0.03) [7]. In addition, RSB was found to reduce opioid consumption by 0.068 mg/kg compared to LAI by 0.23 mg/kg [28]. Further, this group also reportedly had prolonged rescue analgesia at 17.8 min [28].

##### Combined Effect of Local Anesthetics in Combination with Adjuvants in Children

The combined effect of analgesic and local anesthetic in the treatment of POP in children undergoing LA was further investigated. The local anesthetic bupivacaine was administered alone and in combination with dexmedetomidine in children undergoing LA [29]. The two groups received intraperitoneal bupivacaine 0.25% (2 mg/kg) and bupivacaine 0.25% (2 mg/kg) and an adjuvant dexmedetomidine (1 mg/kg). The postoperative visual analog score (VAS) was lower in the group receiving bupivacaine in addition to dexmedetomidine at 2, 4, and 6 h (mean = 3, 3, 3) than in the bupivacaine alone group (mean 4, 5, 4) (*p* > 0.05) [29]. Therefore, the addition of dexmedetomidine to intraperitoneal bupivacaine could provide better pain relief in children undergoing LA.

##### Patient-Controlled Analgesia (PCA) versus Local Anesthesia for Pain in Children

The use of patient-controlled analgesia (PCA) might be more effective for POP in open appendectomy (OA) compared with LA in children [30]. In addition, children treated with PCA during LA might have lower POP. This agrees with the results of Liu et al. [31], who examined various pain management strategies, including local anesthesia at incision sites, IV-opioids via PCA, and prescribed dosing of IV-ketorolac and oral paracetamol/hydrocodone. It appears that when multimodal therapy was used, the level of severe pain was likely reduced (*p* > 0.001). In addition, it has been suggested that the use of morphine via PCA, local anesthetics, and NSAIDs (nonsteroidal anti-inflammatory drugs) may be effective in relieving pain during LA. In contrast, intravenous analgesia does not appear to be significantly different from the control group POP after LA [32].

##### Effect of Administering Local Anesthesia/Analgesia (Pre- and Intraoperative) in Children

Compared to analgesics, there are also different local anesthetic strategies for the treatment of POP after LA in children. Lidocaine, a potent regional anesthetic with anti-inflammatory and analgesic properties, was used to relieve pain in children after LA [23]. In this study, 1.5 mg/kg lidocaine was administered 5 min before induction of general anesthesia, followed by intraoperative administration of 1.5 mg/kg/hour. It was reported that lidocaine could produce a sustained analgesic effect as the time to request first analgesia was reported as {median of 55 (interquartile range (IQR): 40–110) minutes compared with the control group {median of 40.5 (IQR: 28–65)} minutes (*p* = 0.05) [23]. However, the effect of lidocaine on POP was temporary and therefore had no effect on opioid use in the first 24 h after discontinuation of lidocaine. Lidocaine consumption was reported to be lower in the intervention group (32.5 mL) than in the control group 35.0) [33].

##### Effect of Intraperitoneal Administration of Local Anesthetic versus Normal Saline in Children

Twenty mL of bupivacaine spray (0.25% or 1.25%) was injected into the right iliac fossa and the visceral and parietal peritoneum of the pelvis, followed by 20 mg/kg paracetamol (orally) every 24 h or 15 mg/kg paracetamol (IV) to improve treatment POP [34]. With regard to POP, intraperitoneal local anesthesia might offer no clinical benefit in children. There appears to be no significant difference between the treatment (*p* = 0.80) and the control (*p* = 0.89) groups, respectively. They also argued that for successful regional anesthesia, local anesthetics must be placed near the target nerves without risk of disturbing the intended nerves or adjacent areas. Therefore, TAP blockade could be a successful technique in which a local anesthetic is injected into the transverse abdominal plane to block the flow of nerve fibers to the anterior abdominal wall. In addition, UGTAP blockade has been used in children undergoing LA for POP. Port sites were infiltrated with ropivacaine and IV-PCA morphine and paracetamol was recommended [7]. A significant reduction in median pain score was observed for the TAP group at the ward level of 0 compared to 2 (*p* = 0.03) in the control group. Therefore, TAP block could increase the duration of anesthesia by an average of 14 min, which is unlikely to provide a clinically significant advantage over general anesthesia in children undergoing LA.

##### Ultrasound-Guided Quadratus Lumborum Block versus Transversus Abdominis Plane Block in Children

The analgesic efficacy of the TAP blockade procedure was compared with UGQLB. VAS was measured after 2, 4, 6, 8, 12, and 24 h [35]. At the same concentration of levobupivacaine (0.25%), mean VAS-values in the first four hours after surgery were highly statistically significant (*p* < 0.001) and lower in the UGQLB group than in the TAP-block group. However, in the remaining time intervals, there was no statistically significant difference in pain scores between the two groups. UGQLB could therefore provide longer and more effective pain control than TAP -block in children undergoing LA. Again, Maloney et al., [36] also compared UGBRSB (ultrasound-guided bilateral rectal sheath blockade) with local analgesics only and found that UGBRSB had a longer analgesic effect compared with patients receiving local analgesics only. Evidently, patients with UGRSB had lower preliminary (0.38 vs. 2.38; *p* < 0.0001) and mean scores (1.3 vs. 1.8; *p* < 0.015). Therefore, UGBRSB might be a practical preference for POP management in children for solo incision laparoscopic surgery.

##### Effect of Local Anesthetics on Primary and Secondary Outcomes in Children Undergoing Laparoscopic Appendectomy

The use of a 30 °C lens improved coaxial visualization. To achieve technological parity with the LAP-A, a stapler mechanism was used that required expansion of the 5-mm port to a 12-mm port. POP and other indicators were not statistically different from each other at the time of hospitalization or during the follow-up period, according to the results [37]. Similarly, LA is also compared with open appendectomy in terms of primary and secondary outcomes such as nausea, vomiting, hospitalization, and postoperative pain. Two studies with contrasting results were found discussing LA and open appendectomy [38,40]. Wilson et al. [38] advocated long-term analgesia via LA, while Schmelzer et al., [40] indicated that patients need concurrent analgesic prescriptions to ensure that children receive adequate postoperative analgesia both in the hospital and at home. Liu et al. [27] studied the effect of LA and open appendectomy in 115 children. The study concluded that LA was effective in reducing POP and other postoperative outcomes, while LA also resulted in children being discharged the same day [42]. Results showed that of the 207 participants, only 11.1% of children were readmitted due to pain, nausea, or vomiting. However, Applegate et al. [43] concluded that POP and other postoperative outcomes depend on the intensity of the disease, i.e., inflammation, systemic diseases, and their treatment have multiple effects on pain and recovery. Accordingly, Tomecka et al. [31] also concluded that POP is common in children undergoing LA and is usually treated with local anesthesia and emergency analgesia.

### 5.4. Discussion

To our knowledge, the evidence for general pain management in children is unclear because there is no evidence on which medications to use; in fact, postoperative pain management in pediatric surgery is often ineffective. Laparoscopic appendectomy is not the preferred method in children compared with open appendectomy, but it was the most commonly used in some countries. Moreover, it is a technique that transforms a low-risk operation into an intermediate-risk operation from a hemodynamic point of view.

Therefore, this study provides evidence for pain management in this population. Multimodal analgesia appears to be the most commonly used strategy for pain relief in children after laparoscopic appendectomy. This strategy could help reduce the extent of severe pain. However, the multimodal technique of postoperative pain relief in children should include agents with different mechanisms of action: an opioid with an adjuvant and a drug with peripheral analgesic and anti-inflammatory effects (NSAIDs). However, the use of drugs such as morphine and fentanyl carries the risk of serious adverse effects related to respiratory problems, micturition disorders, diuresis, constipation, and the development of dependence. Therefore, in pediatric patients, multimodal therapy should include care, emotional support, and a sense of security. The sedative and anxiolytic effects would be synergistic, could reduce pruritus and psychomotor agitation, and facilitate falling asleep and resting. It is necessary to ensure adequate pain control in pediatric patients even after they leave the medical facility. There is the need for caregivers to be familiarized with the children’s pharmacotherapy regimen. However, whether it is advisable to administer another dose of the drug should not be judged by a caregiver who does not experience pain himself and has not undergone the procedure and therefore does not know the actual pain intensity. Further, the psychological and behavioral aspect is important. The way a child is cared for, the feeling of safety, the presence of a parent, and the speed of return home might have a significant impact on the patient’s well-being and the course of convalescence.

Toward the end of the surgical procedure, administration of dezocin may relieve pain and restlessness. Ketorolac appears to have a more potent analgesic effect compared with acetaminophen and diclofenac sodium. The addition of an adjuvant (e.g., dexmedetomidine) to the local anesthetic may enhance its effect. The use of PCA was found to be more effective than the exclusive use of LA in the treatment of pain in children. Intraperitoneal use of local anesthetics may not be of clinical benefit for postoperative pain in children. Ultrasound-guided quadratus lumborum blockade appeared to be more effective than tranversus blockade 4 h postoperatively in children. Ultrasound-guided rectal sheath blockade also had a longer analgesic effect than local anesthetic administration alone. Therefore, children who experience pain after laparoscopic appendectomy should be treated with long-term analgesia.

In agreement with similar studies, a review has shown that the TAP block procedure reduces the need for opioids 6–8 h postoperatively [36]. Bergmans et al. [44] performed an evaluation of pain relief after TAP block procedure and concluded that it is part of a multimodal approach to pain management in children undergoing abdominal surgery, which is consistent with our findings. To support this fact, the ASA recommended that multimodal approaches should include round-the-clock treatment with acetaminophen, nonselective or selective COX-2 inhibitory NSAID, and regional blockade with local anesthetics [45]. However, it is necessary to control the inflammatory factors in the periphery because the surgical intervention itself causes an increase in inflammation, which is the cause of the intervention. This could be the plausible explanation why ketorolac and diclofenac sodium have a better effect than paracetamol. The latter (paracetamol) acts mainly on the central system by blocking central peroxidases; it is not a sufficient anti-inflammatory agent.

Dezocin has been shown to relieve pain in this study. In addition, from the literature, its analgesic efficacy was comparable to that of morphine and it enhanced the respiratory depression of morphine and also produced a dramatic increase in analgesia, suggesting an additive effect [44]. Dexmedetomidine was an adjuvant that enhanced the effect of local anesthesia in this study. Sarvesh et al. [46] also found that the addition of dexmedetomidine to US-TAP blockade procedures was associated with lower opioid consumption within 24 h. Ketorolac was found to be a more effective analgesic compared with acetaminophen and diclofenac sodium, both NSAIDs. However, in another study, the consumption of ketorolac was found to be different at US-TAP and US-OSTAP [21]. Therefore, further quantification is needed.

The use of PCA was found to be more effective in treating pain in children than the exclusive use of LA. This is consistent with the findings of Alsharari et al. [47], who claimed that PCA was an effective strategy for pain relief in adults. Lidocaine appeared to have a transient analgesic effect. This may be due to the fact that the drug is designed to cause temporary loss of sensory, motor, and autonomic functions when injected or applied near nervous tissue [48]. Although intraperitoneal use of local anesthetics for postoperative pain in children may have no clinical benefit, on the contrary, it has been found to reduce total opioid consumption and pain score throughout the hospital stay in adults [47]. Ultrasound-guided quadratus lumborum blockade was found to be more effective against pain than tranversus abdominal blockade 4 h postoperatively in children. In addition, ultrasound-guided rectal sheath blockade was found to have a longer analgesic effect than the use of local anesthetics alone. However, in adults, analgesic requirements and cumulative daily opioid consumption were higher with US-TAP blockade than with quadratus lumborum blockade [21]. Similarly, less pain was felt with ultrasound-guided blockade of the erector spinae than with ultrasound-guided oblique subcostal transversus abdominis plane [21]. In this study, it was recommended that laparoscopic appendectomy be treated with long-term analgesia. In addition, laparoscopic appendectomy was known to be a low-risk operation [48]; however, the desire for a multimodality approach to treatment has been shown to be essential [49]. However, it is necessary to control inflammatory factors in the periphery, the surgical procedure itself produces an increase in inflammation, which is the cause of the procedure. This is confirmed by the conclusions on the superiority of ketorolac and diclofenac sodium over acetaminophen (paracetamol). The latter (acetaminophen) acts mainly on the central system by blocking central peroxidases; it is not a sufficient anti-inflammatory agent. This clearly indicates the need for long-term analgesia. Therefore, concomitant prescription of analgesics is necessary to ensure that they receive adequate analgesia both in the hospital and at home. However, postoperative outcomes could depend on the intensity of the disease and the particular treatments that could affect the results.

Multimodal analgesia refers to the process of applying analgesics to multiple sites in the pain pathways with the goal of targeting different receptors within the pain pathways, thereby reducing potential side effects [50,51]. Notably, non-pharmacological complementary/alternative medicine therapies are also used in a multimodal approach [52,53]. The molecular targets and tactics employed in multimodal pain management are designed using the neurophysiology of pain as a guide [52]. As part of a multimodal or a preventive analgesic approach, opioids and non-opioid analgesics in lower doses are used to treat acute and chronic pain in children [52]. Although, postoperative pain can be preemptively prevented or alleviated by the use of opioids, preoperative local anesthetics, or both is one of the most cherished beliefs of anesthesiologists today; it is not so clear whether this is actually the case in adults [52]. Likewise, there is no evidence to support or refute this opinion in children. In a multimodal approach, activation of peripheral nociceptors can be attenuated by administration of anti-inflammatory drugs, local anesthetics, and other agents [52]. In the dorsal horn, nociceptive processing and transmission might be affected by administration of local anesthetics, neuraxial opioids, and α-2-adrenergic agonists (e.g., dexmedetomidine) [52].

Although the mechanism of action is unclear, Alsharari et al. [47] claimed that the addition of dexmedetomidine to local anesthetics during central neuraxial blocks and peripheral nerve blocks may improve the effect of local anesthetics and reduce the need for administration of other analgesics. Dexmedetomidine is known to prolong the effects of local anesthesia by inducing local vasoconstriction. In combination with a local anesthetic, it also has sedative, analgesic, antihypertensive, sympatholytic, and bradycardic effects [53]. PCA is a form of pain management that allows individuals to decide when to receive a dose of analgesic. PCA may be a better method of pain relief than having someone (usually a nurse) give you pain medication. We have found this method to be more effective than using LA alone in treating pain in children. However, it should be used with caution because children may not have the skills and competencies to administer accurate dosing.

The use of local anesthetics injected intraperitoneally into the abdominal cavity has been shown to reduce the need for additional postoperative analgesics and sometimes even the side effects of opiates after surgery. However, the mechanism by which it acts remains unclear. There are several explanations, ranging from sensory blockade of peritoneal pain receptors to anti-inflammatory and analgesic effects of local anesthetics to blockade of the vagal afferent nerve, which transmits sensory visceral information to the central nervous system [54,55,56]. Since the local anesthetic is absorbed into the systemic circulation during an intraperitoneal injection, a central effect similar to that of an intravenous infusion is also assumed [57,58]. However, it remains unclear whether the mechanism of action of intraperitoneally injected local anesthetics is through anti-inflammatory mechanisms intraperitoneally, locally somatosensory, or through their systemic absorption and central action [59]. A local anesthetic injected anterior to the quadratus lumborum muscle and posterior to the transverse fascia probably spreads into the thoracic paravertebral space, posterior to the lateral arch and medial and ligaments of the diaphragm, along the internal thoracic fascia and blocks the inferior thoracic sympathetic trunk and somatic nerves [60]. In addition to the current anatomic and clinical literature, two recent studies support this mechanism of action in blocking the anterior quadratus lumborum [22,61,62,63]. Although the mechanism of action of rectus sheath blockade remains unclear, there is evidence that postoperative release of the proinflammatory cytokine IL-6 is increased in patients after general and spinal anesthesia [39]. This increase in IL-6 levels may be correlated with sleep-related changes in pathologic or other conditions.

#### Limitations

Similar to other reviews, there are some limitations in accessing the literature on the management of POP in children undergoing LA. There were very few randomized controlled trials, while most of the studies found were either retrospective or prospective in design and even limited to a single institution. Almost all studies reported only POP in children, or none reported preoperative pain, which is very important for determining the efficacy of the intervention used in this study. Dealing with children in particular is very difficult, especially when it comes to expressing and assessing chronic pain and feelings through self-assessment of pain scores. However, trained personnel familiar with objective instruments (the FLACC scale measures objective parameters) can measure them well, although it is necessary to ensure adequate pain control in pediatric patients even after they leave the medical facility. Nurses and other caregivers should be informed about the pharmacotherapy of a young patient. However, the appropriateness of administering another dose of the drug should not be judged by a caregiver who does not experience pain himself and has not undergone the procedure and therefore does not know the actual intensity of pain. In addition, communication with the children and parents themselves is an important task that may affect the overall outcome of any study. Other limitations that might affect the overall outcome of the study are that we cannot be sure that the estimated effects are due to the treatment and not to other factors.

## 6. Conclusions

Laparoscopic appendectomy is preferred by clinicians compared with open appendectomy; however, it is also necessary to treat POP in children. Although multimodal technique is optimal for analgesia unless contraindications are present, it proves to be effective alongside efficient surgical technique such as UGBRSB, UGQLB, and UGTAP block and can greatly affect POP in children. Dexmedetomidine has been shown to be an effective adjuvant that can enhance the effect of LA. However, there is limited evidence that POP supports treatment with these interventions. Larger RCTs are needed to gather better evidence for POP in children undergoing LA.

## Figures and Tables

**Table 1:** Characteristics of the included studies.

No	Authors	Age (Mean Age and/or Range, Ratio)	Gender	Pain Assessment Scale	Sample Size	Design	Surgical Infiltration Solutions	Drug Used/Doses	Change Scores
1	Liu et al. [27].	≥6 years (experimental 31(46.3); >6 years 36(53.7); control ≥ 6 years 20(41.7); >6 years 28(58.3).	Male (experimental *n* = 36{53.7; control *n* = 27 {56.25); female (experimental *n* = 31{46.27; control *n* = 21 {43.75).	VAS	115	Clinical trial	Not reported	Not reported	Postoperative pain appeared to be significantly lower in the experimental group (2.19 ± 0.34) compared with the control group (3.56 ± 0.37).
2	Kaszynski et al. [38].	5–17 years	Male (*n* = 49, 3.99%); Female (*n* = 49, 69.01%).	FLACC & NRS	71	RCT	IV-lidocaine 1.5 mg/kg bolus 5-min before induction and 1.5 mg/kg/hour intraoperative	Pre-medication IV-midazolam 0.05 mg/kg. Induction analgesia 2. Fentanyl 3 mcg/kg; followed by 2 mcg/kg before incision. Acetaminophen 15 mg/kg and metamizole 10 mg/kg (under anesthesia).1 mcg/kg as maintenance dose. Postoperative Acetaminophen 15 mg/kg 6-hourly and metamizole 10 mg/kg 8-hourly. Nalbuphine (0.1 mg/kg initial dose).	No changes in the first 24 h with cumulative morphine dosing. Lidocaine consumption was lower in the intervention group (32.5 mL) than in the control group (35.0 mL). Time to request first analgesia was prolonged in the intervention group {median 55 (IQR: 40–110) minutes. Compared to the control group {median 40.5 (IQR: 28–65).
3	Alkhoury et al. [24].	2–19 years	not reported	not reported	207	Prospective cohort study	Not reported	Not reported	Equivalent operative duration, i.e., average 23 min.
4	Applegate et al. [26].	NA	NA	FLACC, verbal score (0–10), or FACES	180	Retrospective	NA	Not specified	Those with acute cases received more analgesia after the operation (132.6; 105.8 to 176.1 mcg/kg compared to 93.8; 68.9 to 130.8 mcg/kg; *p* < 0.001).
5	Majeed et al. [39].	7.41 ± 2.73	Male (*n* = 85, 70.83%); Female (*n* = 35, 29.17).	FPS-R	120	Clinical trial	NA	Postoperative Group A: IV-paracetamol 15 mg/kg 6-hourly Group B: diclofenac 2 mg/kg rectally twice daily. Group C: IV-ketorolac 0.5 mg/kg daily.	60-min post-operative 72.5% of Ketorolac group scores 0. 62.5% of Diclofenac group scores 2; and 37.5% of paracetamol group scores 6.
6	Sandeman et al. [7].	7–16 years	NA	VAS	93	RCT	0.5 mL/kg of 0.2% ropivacaine	Intraoperative IV-fentanyl 1 mcg/kg Postoperative IV-morphine bolus 15 mcg/kg with 5-min lockout time Paracetamol 15 mg/kg	Median pain scores for the intervention group in the recovery unit was 0 compared to the 2, (95% CI 0–3, *p* = 0.03)
7	Maloney et al. [34].	LAI group 11.27(3.4); RSB 11.65(3.3)	Male (*n* = 167, 60.73%); Female (*n* = 108, 39.27%)	FACES age or VAS	275	Retrospective	0.25% 1 mL/kg or 0.5% bupivacaine 0.5 mL/kg	Not reported	There was a significant reduction in the consumption of morphine in children who underwent RSB 0.068 mg/kg (±0.007) as compared to the LAI group 0.226 mg/kg (±0.009). No requirement for narcotic during the surgery intra-operatively for RSB group while those receiving local infiltration receivedsome narcotics (*p* < 0.0001; 95% CI 1.41, 1.82). RSB group have a prolonged time to rescue analgesia 17.8 min compared to local infiltration group 58.93 min ± 8.15 versus 41.56 min ± 4.29; *p* = 0.047) Post-operative opioid consumption significantly reduced in RSB group as compared to the local infiltration group (0.04 mg/kg morphine ± 0.005 versus 0.06 mg/kg ± 0.006; *p* = 0.024).
8	Till et al. [26].	Mean age 9.53 years	Both genders were similar as reported.	Not reported	90	Prospective	Not reported	Postoperative Piritramide 0.05 mg/kg with a maximum of 4-boluses within 4-h.	Laparoscopic appendectomy has been reported to decrease pain. It is associated with a decreased needfor analgesics.
9	Tomecka et al. [31].	12.8 (3.8)	Male (*n* = 101, 54.3%; Female (*n* 85, 45.7%).	FLACC, or Wong Baker pain scale, and NRS.	186	Retrospective	Not reported	Intraoperative Fentanyl 1.5–3.0 mcg/kg. Morphine (dose not reported). Postoperative Acetaminophen (dose not reported).	Median postoperative pain was reported in 5% of children; 25% had a median pain score of 4. No association between the use of PCA and postoperative pain.
10	Schmelzer et al. [40].	9.5 ± 3.9	Male (*n* = 138, 61.88%); Female (*n* = 85, 38.12%).	Not reported	223	Retrospective	Not reported	Postoperative Unspecified analgesia	Mean operating room time seem to be longer for the laparoscopic group (62 versus 42-min; *p* < 0.0001). The pain medication was equally less for this group (0.8 versus 1.9 days; *p* = 0.004). There areno differences in postoperative complications for the two groups.
11	Liu et al. [30].	11.0 ± 2.8	Male (*n* = 133, 64.6%); Female (*n* = 73, 35.4%)	NA	206	Retrospective	Bupivacaine 0.25%	Intraoperative IV-fentanyl 2 mcg/kg, morphine 0.1 mg/kg, ketorolac 0.5 mg/kg Postoperative IV-Morphine 0.05 mg/kg up to three; doses After admission to in-patient unit, morphine 0.02 mg/kg administered bolus via PCA after every 10-min to a maximum of 0.35 mg/kg 4-hourly. IV-Ketorolac 0.5 mg/kg 6-hourly × 4 doses	24-h postoperative: 24-patients (11.7% [95% CI 7.8–17.0]) reported substantial pain. The number of subjects reporting at least one episode of severe pain > 6 in the first, second, and third 24-h periods was 31 (15%), 14 (6.8%), and 9 (4.4), respectively. The number of participants reporting at least one episode of moderate pain (4–6) was 77(37%), 19(9%), and 21(10%), respectively. Patients needing more than 0.25 mg/kg of morphine in the first 24-h was higher in the group with generalized peritonitis (43 of 80 [54%] compared to those with peritonitis and simple appendectomy (43 of 126 [34%], p = 0.006). Postoperative pain decreases at day 3 and 4.
12	Sola et al. [29].	IVA and PCA 10.9 ± 4.0 PCA only 9.6 ± 4.0	Male (*n* = 55, 67.1); Female (*n* = 27, 32.9%)	VAS	82	RCT	Not clearly stated	Postoperative Morphine 20 mcg/kg/hour continuous IV for 24-h. Hydromorphone 4 mcg/kg continuous infusion for 24-h IV-ketorolac 0.5 mg/kg 6-hourly for 5 days	No statistically significant difference between for the amount of analgesics (oral) for the two groups (2.8 ± 2.4 versus 2.9 ± 2.5; *p* = 0.88). Transition time from PCA to ral analgesics (76.4 ± 32.5 versus 86.7 ± 49.3 h; *p* = 0.73) for those receiving IVA and non-IVA groups.
13	Perez et al. [41].	2.9–15.7	Male (*n* = 25, 50%) Female (*n =* 25, 50%).	NA	50	RCT	Not reported	Not reported	No differences in hospital length of stay for the two groups to that of analgesics.
14	Elnabtity et al. [33]	Bupivacaine group: 10.75(1.84); Bupivacaine + dexmedetomidine group: 11.53(1.75).	Male (*n* = 30, 57.69%); Female (*n* = 22, 42.31).	VAS	52	RCT	Bupivacaine 0.25%, at a dose of 2 mg/kg, and 5 mLof normal saline; dexmedetomidine 1 mcg/kg (diluted in normal saline) Port sites infiltration (4 mLlignocaines 1% at maximum dose of 3 mg/kg).	Pre-medication Midazolam 0.05 mg/kg intravenously. Induction analgesia Fentanyl 2 mcg/kg; IV-paracetamol 15 mg/kg Postoperative IV-paracetamol 15 mg/kg 8-hourly. IV-pethidine 1 mg/kg to a maximum of 4-doses.	VAS score at 2, 4, and 6 h for bupivacaine plus dexmedetomidine: median (range) 3(1–5), 3(1–7), and 3(1–8); bupivacaine only group: 4(1–7), 5(1–7), and 4(2–7); *p* = 0.04, 0.02, and 0.03).
15	Hamill et al. [25].	8–14 years	Male (*n* = 102, 58.3%)) Female (*n* = 73, 41.7%)	FPS-R	175	RCT	20 mL of 0.25% of 0.125% bupivacaine according to age or 20 mLof NaCl.	Intraoperative Morphine 0.3 mg/kg, fentanyl 2 mcg/kg, paracetamol 15 mg/kg and parecoxib 1 mg/kg up to a maximum of 40 mg. Postoperative Paracetamol 20 mg/kg 6-hourly for 24-h. Tramadol when necessary.	Overall pain scores at rest for the intervention group −0.28 (SE 0.501, *p* = 0.80); and 0.004 (SE 0.028, *p* = 0.89) for the interaction with IPLA. No significant differences for the opioid requirement in IPLA for the time to first and time in PACU.
16	Ellatif et al. [28].	7–12 years	Male (*n* = 19, 55.9%) Female (*n* = 15, 44.1%).	VAS	34	RCT	0.5 mL/kg of 0.25% levobupivacaine	Intraoperative Fentanyl 0.5 mcg/kg Postoperative 1 mg/kg diclofenac sodium Rescue analgesia IV-Paracetamol 15 mg/kg	QLB group reportedly have significantly lower postoperative VAS score 4-h, lower fentanyl dose, and longer time to rescue analgesia.
17	Hu et al. [32].	4–10 years	NA	FLACC	60	RCT	Remifentanil	Intra-operative Dezocine 0.10 mg/kg for group D. Fentanyl 1.0 μg/kg for group F. Control group receives the same volume of normal saline.	MAP and HR seem to be higher for the fentanyl and normal saline groups compared to dezocin (*p* = 0.05).

VAS: Visual Analogue Scale; IV: Intravenous; RSB: Rectus Sheathe Block; LAI: Local Anesthetic Infiltration; FLACC: Face Legs Activity, Cry, Consolability Scale; RCT: Randomized Controlled Trial; FACES: Numerical age-appropriate visualanalogue scale, FPS-R: Revised Faces Pain Scale; NA: Not Available, NRS: Numeric Pain Rating Scale: CI: Confidence Interval: PCA: Patient Controlled Analgesia, IQR: Interquartile range; IVA: Intravenous acetaminophen; PACU: Post Anesthesia Care Unit; IPLA: Intraperitoneal Local Anesthetics; QLB: Quadratus Lumborum Block, MAP: Mean Arterial Pressure; HR: Heart Rate.

## Data Availability

The data that support the findings of this study are available from the corresponding author upon reasonable request.

## Supplementary Materials

The following are available online at https://www.mdpi.com/article/10.3390/healthcare11060870/s1, Supplementary File S1: Search strategy, Supplementary File S2: Studies ineligible following full text review, Supplementary File S3: Characteristics of the included studies.
