# Supplementary material for: Postoperative Pain Management in Children Undergoing Laparoscopic Appendectomy: A Scoping Review
Source: Healthcare (Basel). 2023 Mar 16;11(6):870. doi: 10.3390/healthcare11060870 (PMC10048283; doi:10.3390/healthcare11060870)
Supplement: Supplementary file 1 [file healthcare-11-00870-s001.zip › healthcare-2187150-supplementary.pdf]

Supplementary File S1: Search strategy

Medline (PubMed)

Searched on August 24, 2021

Search	Query
1	“Children and postoperative pain management in appendectomy”
2	“Laparoscopic appendectomy in children”
3	“Postoperative pain management in children undergoing laparoscopic appendectomy”
1465 results	

Cochrane Library

Searched on August 24, 2021

Search	Query
1	“Children and postoperative pain management in appendectomy”
2	“Laparoscopic appendectomy in children”
3	“Postoperative pain management in children undergoing laparoscopic appendectomy”
20 results	

Clinicaltrials.gov

Searched on August 24, 2021

Search	Query
1	“Children and postoperative pain management in appendectomy”
2	“Laparoscopic appendectomy in children”
3	“Postoperative pain management in children undergoing laparoscopic appendectomy”
17 results	

Google Scholar

Searched on August 24, 2021

Search	Query
1	“Children and postoperative pain management in appendectomy”
2	“Laparoscopic appendectomy in children”
3	“Postoperative pain management in children undergoing laparoscopic appendectomy”
133 results	

Embase (Elsevier)

Searched on August 24, 2021

Search	Query
1	“Children and postoperative pain management in appendectomy”
2	“Laparoscopic appendectomy in children”
3	“Postoperative pain management in children undergoing laparoscopic appendectomy”
5319 results	

Supplementary File S2: Studies ineligible following full text review

Duplicate: (duplicate/exact copy; duplicate/same information).

Ineligible concept: not inclusive of all dimensions of POP in children after LA.

Ineligible type of study: Wrong outcome, systematic reviews, scoping review, editorial, opinion.

1. J. K. Hamill, A. Liley, and A. G. Hill, "Sprayed Intraperitoneal Local Anesthetic for Laparoscopic Appendectomy in Children Randomized Controlled Trial."

Reason for exclusion: Duplicate (similar information).

2. M. R. Vahdad, R. Troebs, M. Nissen, L. B. Burkhardt, S. Hardwig, and G. Cernaianu, "Laparoscopic appendectomy for perforated appendicitis in children has complication rates comparable with those of open appendectomy," *J. Pediatr. Surg.*, vol. 48, no. 3, pp. 555–561, 2013, doi: 10.1016/j.jpedsurg.2012.07.066

Reason for exclusion: Ineligible concept.

3. O. Palabiyik and G. Demir, "Chronic Pain after Open Appendectomy and Its Effects on Quality of Life in Children Aged 8 – 18 Years," vol. 2021, 2021

Reason for exclusion: Ineligible concept.

4. W. M. Al-sadek, S. N. Rizk, and M. A. Selim, "Ultrasound guided transversus abdominis plane block in pediatric patients undergoing laparoscopic surgery," *Egypt. J. Anaesth.*, pp. 1–6, 2014, doi: 10.1016/j.egja.2014.01.011

Reason for exclusion: Ineligible concept.

5. I. Batko, B. K. Kościelniak, I. Al-mutari, and K. Kobylarz, "Benefits of ultrasound-guided transversus abdominis plane block for open appendectomy in children," vol. 49, no. 3, pp. 198–203, 2017.

Reason for exclusion: Ineligible concept.

6. E. M. Mahdi, S. Ourshalimian, C. J. Russell, A. K. Zamora, and L. I. Kelley-quon, "Less postoperative opioids associated with decreased duration of stay for children with perforated appendicitis," *Surgery*, pp. 1–6, 2020, doi: 10.1016/j.surg.2020.04.060

Reason for exclusion: Ineligible concept.

7. D. A. Freilich, C. S. Houck, P. M. Meier, C. C. Passerotti, A. B. Retik, and H. T. Nguyen, “The effectiveness of aerosolized intraperitoneal bupivacaine in reducing postoperative pain in children undergoing robotic-assisted laparoscopic pyeloplasty,” 2008, doi: 10.1016/j.jpurol.2008.04.006

Reason for exclusion: Ineligible concept.

8. J. Dingemann, M. Metzelder, J. F. Kuebler, B. Ure, and O. Article, “Laparoscopy for suspected appendicitis in children: May a macroscopically normal appendix be left in situ?,” *Eur. J. Pediatr. Surg.*, vol. 19, no. 3, pp. 153–156, 2009, doi: 10.1055/s-0029-1202248.

Reason for exclusion: Ineligible concept.

9. Minneci PC, Hade EM, Lawrence AE, Sebastião YV, Saito JM, Mak GZ, Fox C, Hirschl RB, Gadepalli S, Helmrath MA, Kohler JE, Leys CM, Sato TT, Lal DR, Landman MP, Kabre R, Fallat ME, Cooper JN, Deans KJ; Midwest Pediatric Surgery Consortium. Association of Nonoperative Management Using Antibiotic Therapy vs Laparoscopic Appendectomy With Treatment Success and Disability Days in Children With Uncomplicated Appendicitis. JAMA. 2020 Aug 11;324(6):581-593. doi: 10.1001/jama.2020.10888. PMID: 32730561; PMCID: PMC7385674.

Reason for exclusion: Ineligible concept.

10. Patkova B, Svenningsson A, Almström M, Eaton S, Wester T, Svensson JF. Nonoperative Treatment Versus Appendectomy for Acute Nonperforated Appendicitis in Children: Five year Follow Up of a Randomized Controlled Pilot Trial. Ann Surg. 2020 Jun;271(6):1030 1035. doi: 10.1097/SLA.0000000000003646. PMID: 31800496.

Reason for exclusion: Ineligible concept.

11. Golebiewski A, Anzelewicz S, Wiejek A, Lubacka D, Czauderna P. A Prospective Randomized Controlled Trial of Single-Port and Three-Port Laparoscopic Appendectomy in Children. J Laparoendosc Adv Surg Tech A. 2019 May;29(5):703-709. doi: 10.1089/lap.2018.0560. Epub 2019 Apr 4. PMID: 30945979.

Reason for exclusion: Ineligible concept.

12: Ali R, Anwar M, Akhtar J. Laparoscopic versus open appendectomy in children: a randomized controlled trial from a developing country. J Pediatr Surg. 2018 Feb;53(2):247 249. doi: 10.1016/j.jpedsurg.2017.11.022. Epub 2017 Nov 14. PMID: 29223666.

Reason for exclusion: Ineligible concept.

13: Shalaby R, Arnos A, Desoky A, Samaha AH. Laparoscopic appendectomy in children: evaluation of different techniques. Surg Laparosc Endosc Percutan Tech. 2001 Feb;11(1):22 7. PMID: 11269551.

Reason for exclusion: Ineligible concept.

14: Lintula H, Kokki H, Vanamo K, Valtonen H, Mattila M, Eskelinen M. The costs and effects of laparoscopic appendectomy in children. Arch Pediatr Adolesc Med. 2004 Jan;158(1):34-7. doi: 10.1001/archpedi.158.1.34. PMID: 14706955.

Reason for exclusion: Ineligible concept.

15: Korlacki W, Dzielicki J. Laparoscopic appendectomy for simple and complicated appendicitis in children--safe or risky procedure. Surg Laparosc Endosc Percutan Tech. 2008 Feb;18(1):29-32. doi: 10.1097/SLE.0b013e31815b2de0. PMID: 18287979.

Reason for exclusion: Ineligible concept.

16: Wu K, Yang L, Wu A, Wang J, Xu S, Zhao H, Huang Z. Single-site laparoscopic appendectomy in children using conventional instruments: a prospective, randomized, control trial. Pediatr Surg Int. 2015 Feb;31(2):167-71. doi: 10.1007/s00383-014-3636-1. Epub 2014 Nov 9. PMID: 25381588.

Reason for exclusion: Ineligible concept.

17: Nataraja RM, Panabokke G, Chang AD, Mennie N, Tanny ST, Keys C, Cheng W, Pacilli M, Ferguson P. Does peritoneal lavage influence the rate of complications following pediatric Laparoscopic Appendicectomy in Children with Complicated Appendicitis? A Prospective Randomized Clinical Trial. J Pediatr Surg. 2019 Dec;54(12):2524-2527. doi: 10.1016/j.jpedsurg.2019.08.039. Epub 2019 Aug 30. PMID: 31519360.

Reason for exclusion: Ineligible concept.

18: Simon P, Burkhardt U, Sack U, Kaisers UX, Muensterer OJ. Inflammatory response is no different in children randomized to laparoscopic or open appendectomy. J Laparoendosc Adv Surg Tech A. 2009 Apr;19 Suppl 1:S71-6. doi: 10.1089/lap.2008.0155.supp. PMID: 18999981.

Reason for exclusion: Ineligible concept.

19: Little DC, Custer MD, May BH, Blalock SE, Cooney DR. Laparoscopic appendectomy: An unnecessary and expensive procedure in children? J Pediatr Surg. 2002 Mar;37(3):310-7. doi: 10.1053/jpsu.2002.30841. PMID: 11877640.

Reason for exclusion: Ineligible concept.

20: Ostlie DJ. Single-site umbilical laparoscopic appendectomy. Semin Pediatr Surg. 2011 Nov;20(4):196-200. doi: 10.1053/j.sempedsurg.2011.05.003. PMID: 21968154.

Reason for exclusion: Ineligible concept.

21: Fraser JD, Aguayo P, Leys CM, Keckler SJ, Newland JG, Sharp SW, Murphy JP, Snyder CL, Sharp RJ, Andrews WS, Holcomb GW 3rd, Ostlie DJ, St Peter SD. A complete course of intravenous antibiotics vs a combination of intravenous and oral antibiotics for perforated appendicitis in children: a prospective, randomized trial. J Pediatr Surg. 2010 Jun;45(6):1198-202. doi: 10.1016/j.jpedsurg.2010.02.090. PMID: 20620320.

Reason for exclusion: Ineligible concept.

22: Pogorelić Z, Katić J, Mrklić I, Jerončić A, Šušnjar T, Jukić M, Vilović K, Perko Z. Lateral thermal damage of mesoappendix and appendiceal base during laparoscopic appendectomy in children: comparison of the harmonic scalpel (Ultracision), bipolar coagulation (LigaSure), and thermal fusion technology (MiSeal). J Surg Res. 2017 May 15;212:101-107. doi: 10.1016/j.jss.2017.01.014. Epub 2017 Jan 28. PMID: 28550895.

Reason for exclusion: Ineligible concept.

23: Fawkner-Corbett D, Jawaid WB, McPartland J, Losty PD. Interval appendectomy in children clinical outcomes, financial costs and patient benefits. Pediatr Surg Int. 2014 Jul;30(7):743-6. doi: 10.1007/s00383-014-3521-y. Epub 2014 May 30. PMID: 24874344.

Reason for exclusion: Ineligible concept.

24: Saha N, Saha DK, Rahman MA, Islam MK, Aziz MA. Comparison of post operative morbidity between laparoscopic and open appendectomy in children. Mymensingh Med J. 2010 Jul;19(3):348-52. PMID: 20639825.

Reason for exclusion: Ineligible concept.

25: Akın M, Erginel B, Yıldız A, Bayraktar B, Yanar F, Karadağ CA, Sever N, Dokucu AI. Role of dissemination of microorganisms during laparoscopic appendectomy in abscess formation. Ulus Travma Acil Cerrahi Derg. 2014 Jan;20(1):28-32. doi: 10.5505/tjtes.2014.40359. PMID: 24639312.

Reason for exclusion: Ineligible concept.

26: St Peter SD, Tsao K, Spilde TL, Holcomb GW 3rd, Sharp SW, Murphy JP, Snyder CL, Sharp RJ, Andrews WS, Ostlie DJ. Single daily dosing ceftriaxone and metronidazole vs standard triple antibiotic regimen for perforated appendicitis in children: a prospective randomized trial. J Pediatr Surg. 2008 Jun;43(6):981-5. doi: 10.1016/j.jpedsurg.2008.02.018. PMID: 18558169; PMCID: PMC3082440.

Reason for exclusion: Ineligible concept.

27: Badawy H, Eid A, Dawood W, Hanno A. Safety and feasibility of laparoscopic appendicovesicostomy in children. J Pediatr Urol. 2013 Aug;9(4):427-31. doi: 10.1016/j.jpurol.2012.05.013. Epub 2012 Jul 7. PMID: 22771193.

Reason for exclusion: Ineligible concept.

28: Goh BK, Chui CH, Yap TL, Low Y, Lama TK, Alkouder G, Prasad S, Jacobsen AS. Is early laparoscopic appendectomy feasible in children with acute appendicitis presenting with an appendiceal mass? A prospective study. J Pediatr Surg. 2005 Jul;40(7):1134-7. doi: 10.1016/j.jpedsurg.2005.03.046. PMID: 16034758.

Reason for exclusion: Ineligible concept.

29: Oka T, Kurkchubasche AG, Bussey JG, Wesselhoeft CW Jr, Tracy TF Jr, Luks FI. Open and laparoscopic appendectomy are equally safe and acceptable in children. Surg Endosc. 2004 Feb;18(2):242-5. doi: 10.1007/s00464-003-8140-y. Epub 2003 Dec 29. PMID: 14691709.

Reason for exclusion: Ineligible concept.

30: Lavonius MI, Liesjärvi S, Ovaska J, Pajulo O, Ristkari S, Alanen M. Laparoscopic versus open appendectomy in children: a prospective randomized study. Eur J Pediatr Surg. 2001 Aug;11(4):235-8. doi: 10.1055/s-2001-17150. PMID: 11558012.

Reason for exclusion: Ineligible concept.

31: Lejus C, Delile L, Plattner V, Baron M, Guillou S, Héloury Y, Souron R. Randomized, single-blinded trial of laparoscopic versus open appendectomy in children: effects on postoperative analgesia. Anesthesiology. 1996 Apr;84(4):801-6. doi: 10.1097/00000542-199604000-00006. PMID: 8638833.

Reason for exclusion: Ineligible concept.

32: Mennie N, Panabokke G, Chang A, Tanny ST, Cheng W, Pacilli M, Ferguson P, Nataraja RM. Are Postoperative Intravenous Antibiotics Indicated After Laparoscopic Appendicectomy for Simple Appendicitis? A Prospective Double- blinded Randomized Controlled Trial. Ann Surg. 2020 Aug;272(2):248-252. doi: 10.1097/SLA.0000000000003732. PMID: 32675537.

Reason for exclusion: Ineligible concept.

33: Hay SA. Laparoscopic versus conventional appendectomy in children. Pediatr Surg Int. 1998 Jan;13(1):21-3. doi: 10.1007/s003830050235. PMID: 9391198.

Reason for exclusion: Ineligible concept.

34: Lintula H, Kokki H, Vanamo K, Antila P, Eskelinen M. Laparoscopy in children with complicated appendicitis. J Pediatr Surg. 2002 Sep;37(9):1317-20. doi: 10.1053/jpsu.2002.34998. PMID: 12194123.

Reason for exclusion: Ineligible concept.

35: Baird R, Ingelmo P, Wei A, Meghani Y, Perez EV, Pelletier H, Auer G, Mujallid R, Emil S, Laberge JM, Puligandla P, Shaw K, Poenaru D. Nebulized analgesia during laparoscopic appendectomy (NALA): A randomized triple-blind placebo controlled trial. J Pediatr Surg. 2019 Jan;54(1):33-38. doi: 10.1016/j.jpedsurg.2018.10.029. Epub 2018 Oct 5. PMID: 30366723.

Reason for exclusion: Ineligible concept.

36: Kim JY, Shin CS, Lee KC, Chang YJ, Kwak HJ. Effect of pressure- versus volume-controlled ventilation on the ventilatory and hemodynamic parameters during laparoscopic appendectomy in children: a prospective, randomized study. J Laparoendosc Adv Surg Tech A. 2011 Sep;21(7):655-8. doi: 10.1089/lap.2011.0051. Epub 2011 Jun 23. PMID: 21699432.

Reason for exclusion: Ineligible concept.

37: Schoenberg MB, Magdeburg R, Kienle P, Post S, Eisser PP, Kähler G. Hybrid transgastric appendectomy is feasible but does not offer advantages compared with laparoscopic appendectomy: Results from the transgastric appendectomy study. Surgery. 2017 Aug;162(2):295-302. doi: 10.1016/j.surg.2017.02.013. Epub 2017 Apr 23. PMID: 28442133.

Reason for exclusion: Ineligible concept.

38: Kang BM, Choi SI, Kim BS, Lee SH. Single-port laparoscopic surgery in uncomplicated acute appendicitis: a randomized controlled trial. Surg Endosc. 2018 Jul;32(7):3131-3137. doi: 10.1007/s00464-018-6028-0. Epub 2018 Jan 16. PMID: 29340826.

Reason for exclusion: Ineligible concept.

39: Stylianos S, Nichols L, Ventura N, Malvezzi L, Knight C, Burnweit C. The "all-in-one" appendectomy: quick, scarless, and less costly. J Pediatr Surg. 2011 Dec;46(12):2336-41. doi: 10.1016/j.jpedsurg.2011.09.029. PMID: 22152877.

Reason for exclusion: Ineligible concept.

40: Erfanian K, Luks FI, Kurkchubasche AG, Wesselhoeft CW Jr, Tracy TF Jr. In- line image projection accelerates task performance in laparoscopic appendectomy. J Pediatr Surg. 2003 Jul;38(7):1059-62. doi: 10.1016/s0022-3468(03)00192-1. PMID: 12861539.

Reason for exclusion: Ineligible concept.

41: Snow HA, Choi JM, Cheng MW, Chan ST. Irrigation versus suction alone during laparoscopic appendectomy; A randomized controlled equivalence trial. Int J Surg. 2016 Apr;28:91-6. doi: 10.1016/j.ijsu.2016.01.099. Epub 2016 Feb 18. PMID: 26912015.

Reason for exclusion: Ineligible concept.

42: St Peter SD, Aguayo P, Fraser JD, Keckler SJ, Sharp SW, Leys CM, Murphy JP, Snyder CL, Sharp RJ, Andrews WS, Holcomb GW 3rd, Ostlie DJ. Initial laparoscopic appendectomy versus initial nonoperative management and interval appendectomy for perforated appendicitis with abscess: a prospective, randomized trial. J Pediatr Surg. 2010 Jan;45(1):236-40. doi: 10.1016/j.jpedsurg.2009.10.039. PMID: 20105610.

Reason for exclusion: Ineligible concept.

43: Perez EA, Piper H, Burkhalter LS, Fischer AC. Single-incision laparoscopic surgery in children: a randomized control trial of acute appendicitis. Surg Endosc. 2013 Apr;27(4):1367-71. doi: 10.1007/s00464-012-2617-5. Epub 2012 Dec 13. PMID: 23239295.

Reason for exclusion: Ineligible concept.

44: Moriguchi T, Machigashira S, Sugita K, Kawano M, Yano K, Onishi S, Yamada K, Yamada W, Masuya R, Kawano T, Nakame K, Mukai M, Kaji T, Ieiri S. A Randomized Trial to Compare the Conventional Three-Port Laparoscopic Appendectomy Procedure to Single-Incision and One-Puncture Procedure That Was Safe and Feasible, Even for Surgeons in Training. J Laparoendosc Adv Surg Tech A. 2019 Mar;29(3):392-395. doi: 10.1089/lap.2018.0195. Epub 2018 Nov 10. PMID: 30418099.

Reason for exclusion: Ineligible concept.

45: Olmi S, Magnone S, Bertolini A, Croce E. Laparoscopic versus open appendectomy in acute appendicitis: a randomized prospective study. Surg Endosc. 2005 Sep;19(9):1193-5. doi: 10.1007/s00464-004-2165-8. Epub 2005 Jul 28. PMID: 16132334.

Reason for exclusion: Ineligible concept.

46: Rızalar S, Özbaş A. Effect of Early Postoperative Feeding on the Recovery of Children Post Appendectomy. Gastroenterol Nurs. 2018 Mar/Apr;41(2):131-140. doi: 10.1097/SGA.0000000000000279. PMID: 29596127.

Reason for exclusion: Ineligible concept.

47: Williams MD, Collins JN, Wright TF, Fenoglio ME. Laparoscopic versus open appendectomy. South Med J. 1996 Jul;89(7):668-74. doi: 10.1097/00007611-199607000-00004. PMID: 8685751.

Reason for exclusion: Ineligible concept.

48: Frutos MD, Abrisqueta J, Lujan J, Abellan I, Parrilla P. Randomized prospective study to compare laparoscopic appendectomy versus umbilical single- incision appendectomy. Ann Surg. 2013 Mar;257(3):413-8. doi: 10.1097/SLA.0b013e318278d225. PMID: 23386239.

Reason for exclusion: Ineligible concept.

49: Khalil J, Muqim R, Rafique M, Khan M. Laparoscopic versus open appendectomy: a comparison of primary outcome measures. Saudi J Gastroenterol. 2011 Jul- Aug;17(4):236 40. doi: 10.4103/1319-3767.82574. PMID: 21727728; PMCID: PMC3133979.

Reason for exclusion: Ineligible concept.

50: St Peter SD, Adibe OO, Iqbal CW, Fike FB, Sharp SW, Juang D, Lanning D, Murphy JP, Andrews WS, Sharp RJ, Snyder CL, Holcomb GW, Ostlie DJ. Irrigation versus suction alone during laparoscopic appendectomy for perforated appendicitis: a prospective randomized trial. Ann Surg. 2012 Oct;256(4):581-5. doi: 10.1097/SLA.0b013e31826a91e5. PMID: 22964730.

Reason for exclusion: Ineligible concept.

51: Lintula H, Kokki H, Vanamo K. Single-blind randomized clinical trial of laparoscopic versus open appendicectomy in children. Br J Surg. 2001 Apr;88(4):510-4. doi: 10.1046/j.1365-2168.2001.01723.x. PMID: 11298617.

Reason for exclusion: Ineligible concept.

52: Russell KW, Rollins MD, Barnhart DC, Mone MC, Meyers RL, Skarda DE, Soukup ES, Black RE, Molitor MS, Stoddard GJ, Scaife ER. Charge Awareness Affects Treatment Choice: Prospective Randomized Trial in Pediatric Appendectomy. Ann Surg. 2015 Jul;262(1):189-93. doi: 10.1097/SLA.0000000000000885. PMID: 25185471.

Reason for exclusion: Ineligible concept.

53: Stringel G, Berezin SH, Bostwick HE, Halata MS. Laparoscopy in the management of children with chronic recurrent abdominal pain. JSLS. 1999 Jul-Sep;3(3):215-9. PMID: 10527334; PMCID: PMC3113158.

Reason for exclusion: Ineligible concept.

54: Blakely ML, Williams R, Dassinger MS, Eubanks JW 3rd, Fischer P, Huang EY, Paton E, Culbreath B, Hester A, Streck C, Hixson SD, Langham MR Jr. Early vs interval appendectomy for children with perforated appendicitis. Arch Surg. 2011 Jun;146(6):660-5. doi: 10.1001/archsurg.2011.6. Epub 2011 Feb 21. PMID: 21339413.

Reason for exclusion: Ineligible concept.

55: St Peter SD, Adibe OO, Juang D, Sharp SW, Garey CL, Laituri CA, Murphy JP, Andrews WS, Sharp RJ, Snyder CL, Holcomb GW 3rd, Ostlie DJ. Single incision versus standard 3-port laparoscopic appendectomy: a prospective randomized trial. Ann Surg. 2011 Oct;254(4):586-90. doi: 10.1097/SLA.0b013e31823003b5. PMID: 21946218.

Reason for exclusion: Ineligible concept.

56: Gollin G, Abarbanell A, Moores D. Oral antibiotics in the management of perforated appendicitis in children. Am Surg. 2002 Dec;68(12):1072-4. PMID: 12516811.

Reason for exclusion: Ineligible concept.

57: Smania MC, Piva JP, Garcia PC. Dexmedetomidine in anesthesia of children submitted to videolaparoscopic appendectomy: a double-blind, randomized and placebo-controlled study. Rev Assoc Med Bras (1992). 2008 Jul-Aug;54(4):308-13. doi: 10.1590/s0104-42302008000400015. PMID: 18719788.

Reason for exclusion: Ineligible concept.

58: Hebebrand D, Troidl H, Spangenberger W, Neugebauer E, Schwalm T, Günther MW. Laparoskopische oder klassische Appendektomie? Eine prospektiv randomisierte Studie [Laparoscopic or classical appendectomy? A prospective randomized study].Chirurg. 1994 Feb;65(2):112-20. German. PMID: 8162812.

Reason for exclusion: Ineligible concept.

59: Lorenz EP, Ehren G, Schmidt M, Soop J, Konradt J. Die laparoskopische Appendektomie als Standardverfahren. Technik und Ergebnisse bei 409 Patienten [Laparoscopic appendectomy as standard procedure. Technique and outcome in 409 patients]. Zentralbl Chir. 1998;123 Suppl 4:97-100. German. PMID: 9880886.

Reason for exclusion: Ineligible concept.

60: Knott EM, Gasior AC, Holcomb GW 3rd, Ostlie DJ, St Peter SD. Impact of body habitus on single-site laparoscopic appendectomy for nonperforated appendicitis: subset analysis from a prospective, randomized trial. J Laparoendosc Adv Surg Tech A. 2012 May;22(4):404-7. doi: 10.1089/lap.2012.0056. PMID: 22577809.

Reason for exclusion: Ineligible concept.

61: Miyano G, Urao M, Lane G, Kato Y, Okazaki T, Yamataka A. Appendiceal stump closure in children with complicated appendicitis: a prospective analysis of endoloops versus endostaples. Asian J Endosc Surg. 2011 Aug;4(3):116-9. doi: 10.1111/j.1758-5910.2011.00091.x. Epub 2011 Jun 20. PMID: 22776274.

Reason for exclusion: Ineligible concept.

62: Bjerrum F, Sorensen JL, Konge L, Lindschou J, Rosthøj S, Ottesen B, Strandbygaard J. Procedural specificity in laparoscopic simulator training: protocol for a randomised educational superiority trial. BMC Med Educ. 2014 Oct 10;14:215. doi: 10.1186/1472-6920-14-215. PMID: 25304939; PMCID: PMC4201735.

Reason for exclusion: Ineligible concept.

63: Romero P, Frongia G, Wingerter S, Holland-Cunz S. Prospective, randomized, controlled trial comparing a tissue adhesive (Dermabond™) with adhesive strips (Steri Strips™) for the closure of laparoscopic trocar wounds in children. Eur J Pediatr Surg. 2011 May;21(3):159-62. doi: 10.1055/s-0030-1270458. Epub 2011 Jan 31. PMID: 21283958.

Reason for exclusion: Ineligible concept.

64: Schurman JV, Cushing CC, Garey CL, Laituri CA, St Peter SD. Quality of life assessment between laparoscopic appendectomy at presentation and interval appendectomy for perforated appendicitis with abscess: analysis of a prospective randomized trial. J Pediatr Surg. 2011 Jun;46(6):1121-5. doi: 10.1016/j.jpedsurg.2011.03.038. PMID: 21683209.

Reason for exclusion: Ineligible concept.

65: Huang MT, Wei PL, Wu CC, Lai IR, Chen RJ, Lee WJ. Needlescopic, laparoscopic, and open appendectomy: a comparative study. Surg Laparosc Endosc Percutan Tech. 2001 Oct;11(5):306-12. doi: 10.1097/00129689-200110000-00004. Erratum in: Surg Laparosc Endosc Percutan Tech 2002 Aug;12(4):following table of contents. PMID: 11668227.

Reason for exclusion: Ineligible concept.

66: Bjerrum F, Sorensen JL, Konge L, Rosthøj S, Lindschou J, Ottesen B, Strandbygaard J. Randomized trial to examine procedure-to-procedure transfer in laparoscopic simulator training. Br J Surg. 2016 Jan;103(1):44-50. doi: 10.1002/bjs.9966. Epub 2015 Oct 29. PMID: 26511775.

Reason for exclusion: Ineligible concept.

67: Yu TC, Hamill JK, Liley A, Hill AG. Warm, humidified carbon dioxide gas insufflation for laparoscopic appendicectomy in children: a double-blinded randomized controlled trial. Ann Surg. 2013 Jan;257(1):44-53. doi: 10.1097/SLA.0b013e31825f0721. PMID: 22824858.

Reason for exclusion: Ineligible concept.

68: Frazee RC, Roberts JW, Symmonds RE, Snyder SK, Hendricks JC, Smith RW, Custer MD 3rd, Harrison JB. A prospective randomized trial comparing open versus laparoscopic appendectomy. Ann Surg. 1994 Jun;219(6):725-8; discussion 728-31. doi: 10.1097/00000658 199406000-00017. PMID: 8203983; PMCID: PMC1243232.

Reason for exclusion: Ineligible concept.

69: Enochsson L, Gudbjartsson T, Hellberg A, Rudberg C, Wenner J, Ringqvist I, Sörensen S, Fenyö G. The Fenyö-Lindberg scoring system for appendicitis increases positive predictive value in fertile women--a prospective study in 455 patients randomized to either laparoscopic or open appendectomy. Surg Endosc. 2004 Oct;18(10):1509-13. doi: 10.1007/s00464-003-9323-2. Epub 2004 Aug 24. PMID: 15791379.

Reason for exclusion: Ineligible concept.

70: Gee K, Ngo S, Burkhalter L, Beres AL. Safety and feasibility of same-day discharge for uncomplicated appendicitis: A prospective cohort study. J Pediatr Surg. 2018 May;53(5):988-990. doi: 10.1016/j.jpedsurg.2018.02.031. Epub 2018 Feb 9. PMID: 29510871.

Reason for exclusion: Ineligible concept.

71: Pruett B, Pruett J. Laparoscopic appendectomy: have we found a better way? J Miss State Med Assoc. 1994 Dec;35(12):347-51. PMID: 7869368.

Reason for exclusion: Ineligible concept.

72: Reiertsen O, Trondsen E, Bakka A, Andersen OK, Larsen S, Rosseland AR. Prospective nonrandomized study of conventional versus laparoscopic appendectomy. World J Surg. 1994 May-Jun;18(3):411-5; discussion 415-6. doi: 10.1007/BF00316823. PMID: 8091783.

Reason for exclusion: Ineligible concept.

73: Pogorelić Z, Silov N, Jukić M, Elezović Baloević S, Poklepović Peričić T, Jerončić A. Ertapenem Monotherapy versus Gentamicin Plus Metronidazole for Perforated Appendicitis in Pediatric Patients. Surg Infect (Larchmt). 2019 Dec;20(8):625-630. doi: 10.1089/sur.2019.025. Epub 2019 May 17. PMID: 31099712.

Reason for exclusion: Ineligible concept.

74: Schaarschmidt K, Kerremanns I, Schleef J, Förster R, Pattyn P, Stratmann U, Willital GH, Scheld HH. Laparoscopic and thoracoscopic surgery in infancy and childhood, the Münster/Gent experience. Technol Health Care. 1996 Mar;3(4):263-71. PMID: 8705401.

Reason for exclusion: Ineligible concept.

75: Schietroma M, Piccione F, Carlei F, Clementi M, Bianchi Z, de Vita F, Amicucci G. Peritonitis from perforated appendicitis: stress response after laparoscopic or open treatment. Am Surg. 2012 May;78(5):582-90. PMID: 22546132.

Reason for exclusion: Ineligible concept.

76: Kotlobovskiĭ VI, Dronov AF, Poddubnyĭ IV, Dzhenalaev BK. Sravnitel'noe izuchenie rezul'tatov lecheniia rasprostranennykh form appendikuliarnogo peritonita u deteĭ, operirovannykh laparoskopicheskim i traditsionnym khirurgicheskim sposobom [Comparative study of surgical and endosurgical treatment of generalized appendicular peritonitis in children]. Khirurgiia (Mosk). 2003;(7):32-7. Russian. PMID: 12926337.

Reason for exclusion: Ineligible concept.

77: Bolla G, Tuzzato G. Post-appendectomy immunologic competence in pediatric age. The results do not always match expectations. Laparoscopy versus laparotomy. Pediatr Med Chir. 2008 Mar-Apr;30(2):89-93. PMID: 18581961.

Reason for exclusion: Ineligible concept.

78: Wille MA, Jayram G, Gundeti MS. Feasibility and early outcomes of robotic- assisted laparoscopic Mitrofanoff appendicovesicostomy in patients with prune belly syndrome. BJU Int. 2012 Jan;109(1):125-9. doi: 10.1111/j.1464-410X.2011.10317.x. Epub 2011 Jul 28. PMID: 21794067.

Reason for exclusion: Ineligible concept.

79: Nakaoka T, Uemura S, Yoshida T, Tanimoto T, Shiokawa C, Harumoto K. Umbilical center insertion method for initial trocar placement in pediatric laparoscopic surgery. Osaka City Med J. 2010 Dec;56(2):21-6. PMID: 21466126.

Reason for exclusion: Ineligible concept.

80: Joshi MR, Shrestha SK, Thapa PB, Koirala U, Bhattarai P, Dongol UM, Singh DR, Sharma SK. Use of percutaneous thread loop to hold the vermiform appendix during laparoscopic appendectomy. Kathmandu Univ Med J (KUMJ). 2007 Jan- Mar;5(1):63-7. PMID: 18603988.

Reason for exclusion: Ineligible concept.

8: Lujan Mompean JA, Robles Campos R, Parrilla Paricio P, Soria Aledo V, Garcia

Ayllon J. Laparoscopic versus open appendicectomy: a prospective assessment. Br

J Surg. 1994 Jan;81(1):133-5. doi: 10.1002/bjs.1800810148. PMID: 8313090.

Reason for exclusion: Ineligible concept.

79: Paya K, Rauhofer U, Rebhandl W, Deluggi S, Horcher E. Perforating appendicitis. An indication for laparoscopy? Surg Endosc. 2000 Feb;14(2):182-4. doi: 10.1007/s004649900096. PMID: 10656957.

Reason for exclusion: Ineligible concept.

80: Pedersen AG, Petersen OB, Wara P, Rønning H, Qvist N, Laurberg S. Randomized clinical trial of laparoscopic versus open appendicectomy. Br J Surg. 2001 1Feb;88(2):200-5. doi: 10.1046/j.1365-2168.2001.01652.x. PMID: 11167866.

Reason for exclusion: Ineligible concept.

81: Beyer-Berjot L, Maggiori L, Birnbaum D, Lefevre JH, Berdah S, Panis Y. A total laparoscopic approach reduces the infertility rate after ileal pouch-anal anastomosis: a 2 center study. Ann Surg. 2013 Aug;258(2):275-82. doi: 10.1097/SLA.0b013e3182813741. PMID: 23360923.

Reason for exclusion: Ineligible concept.

82: St Peter SD, Sharp SW, Holcomb GW 3rd, Ostlie DJ. An evidence-based definition for perforated appendicitis derived from a prospective randomized trial. J Pediatr Surg. 2008 Dec;43(12):2242-5. doi: 10.1016/j.jpedsurg.2008.08.051. PMID: 19040944.

Reason for exclusion: Ineligible concept.

83: Putz A, Bogesits R, Müller W, Werner C. Die laparoskopische Appendektomie als Routineeingriff [Laparoscopic appendectomy as a routine procedure]. Infection. 1993;21 Suppl 1:S54-8. German. doi: 10.1007/BF01710345. PMID: 8314295.

Reason for exclusion: Ineligible concept.

84: Reiertsen O, Larsen S, Trondsen E, Edwin B, Faerden AE, Rosseland AR. Randomized controlled trial with sequential design of laparoscopic versus conventional appendicectomy. Br J Surg. 1997 Jun;84(6):842-7. PMID: 9189105.

Reason for exclusion: Ineligible concept.

85: Montupet P, Alain JL, Chavrier Y, Limone B, Valla JS, Varlet F. Appendicites aiguës et péritonites appendiculaires chez l'enfant. Le traitement coelioscopique [Acute appendicitis and appendiceal peritonitis in children. Celioscopic treatment]. Chirurgie. 1993 1994;119(8):433-5. French. PMID: 7805509.

Reason for exclusion: Ineligible concept.

86: Sarfati MR, Hunter GC, Witzke DB, Bebb GG, Smythe SH, Boyan S, Rappaport WD. Impact of adjunctive testing on the diagnosis and clinical course of patients with acute appendicitis. Am J Surg. 1993 Dec;166(6):660-4; discussion 664-5. doi: 10.1016/s0002 9610(05)80675-0. PMID: 8273845.

Reason for exclusion: Ineligible concept.

87: Borgstein PJ, Gordijn RV, Eijsbouts QA, Cuesta MA. Acute appendicitis—a clear-cut case in men, a guessing game in young women. A prospective study on the role of laparoscopy. Surg Endosc. 1997 Sep;11(9):923-7. doi: 10.1007/s004649900488. PMID: 9294274.

Reason for exclusion: Ineligible concept.

88: Azaro EM, Amaral PC, Ettinger JE, Souza EL, Fortes MF, Alcântara RS, Regis AB, Sousa MM, Fogagnoli WG, do Carmo VM, Galvão TD, Stagliorio EP, Santana PA Jr, Fahel E. Laparoscopic versus open appendicectomy: a comparative study. JSLS. 1999 Oct Dec;3(4):279-83. PMID: 10694074; PMCID: PMC3015367.

Reason for exclusion: Ineligible concept.

89: Ramos JM, Beart RW Jr, Goes R, Ortega AE, Schlinkert RT. Role of laparoscopy in colorectal surgery. A prospective evaluation of 200 cases. Dis Colon Rectum. 1995 May;38(5):494-501. doi: 10.1007/BF02148849. PMID: 7736880.

Reason for exclusion: Ineligible concept.

90: Chao C, Tsai CT, Wu WC. Complete two-handed laparoscopic appendectomy: report of 100 cases. J Formos Med Assoc. 1995 Nov;94(11):679-82. PMID: 8527975.

Reason for exclusion: Ineligible concept.

91: Lamparelli MJ, Hoque HM, Pogson CJ, Ball AB. A prospective evaluation of the combined use of the modified Alvarado score with selective laparoscopy in adult females in the management of suspected appendicitis. Ann R Coll Surg Engl. 2000 May;82(3):192-5. PMID: 10858683; PMCID: PMC2503424.

Reason for exclusion: Ineligible concept.

92: Richter M, Laffer U, Ayer G, Blessing H, Biaggi J, Bruttin JM, Brugger JJ, Liechti J, König W. Wird tatsächlich zu häufig appendektomiert? Resultate der prospektiven Multicenterstudie der Schweizerischen Gesellschaft für Allgemeinchirurgie (SGAC) [Is appendectomy really performed too frequently? Results of the prospective multicenter study of the Swiss Society of General Surgery]. Swiss Surg. 2000;6(3):101-7. German. doi: 10.1024/1023-9332.6.3.101. PMID: 10894009.

Reason for exclusion: Ineligible concept.

93: Sand M, Bechara FG, Holland-Letz T, Sand D, Mehnert G, Mann B. Diagnostic value of hyperbilirubinemia as a predictive factor for appendiceal perforation in acute appendicitis. Am J Surg. 2009 Aug;198(2):193-8. doi: 10.1016/j.amjsurg.2008.08.026. Epub 2009 Mar 23. PMID: 19306980.

Reason for exclusion: Ineligible concept.

94: Browne DS. Laparoscopic-guided appendicectomy. A study of 100 consecutive cases. Aust N Z J Obstet Gynaecol. 1990 Aug;30(3):231-3. doi: 10.1111/j.1479-828x.1990.tb03220.x. PMID: 2147847.

Reason for exclusion: Ineligible concept.

95: Jadallah FA, Abdul-Ghani AA, Tibblin S. Diagnostic laparoscopy reduces unnecessary appendicectomy in fertile women. Eur J Surg. 1994 Jan;160(1):41-5. PMID: 8186313.

Reason for exclusion: Ineligible concept.

96. Arabi, Y. M., Tamim, H. M., Rishu, A. H., Bilotta, F., Caramia, R., & Paoloni, F. P. (2010). Bibliography Current World Literature Vol 23 No 5 October 2010. *J Trauma, 68*, 904-911.

Reason for exclusion: Ineligible concept.

97. Prentice, D. C. (1997). *A correlational study of nurses' assessment of patients' pain using the Post Anesthesia Care Unit Behavioral Pain Rating Scale and patients' self-report of pain* (Doctoral dissertation, Clarkson College).

Reason for exclusion: Ineligible concept.

98. Portfolio, C. P. D. (2020). Free CPD App.

Reason for exclusion: Ineligible concept.

99. Berkenstadt, H., Haviv, Y., Tuval, A., & Shemesh, Y. (2008). ICU care processes. *disasters*, *133*, 1065-1066.

Reason for exclusion: Ineligible concept.

100. AN, T. O. 5. ADMINISTRATION OF ANALGESIC MEDICINES.

Reason for exclusion: Ineligible concept.

101. CARTER, B., Howard, R., Curry, J., Jain, A., Liossi, C., Morton, N., ... & Williams, G. (2012). Good Practice in Postoperative and Procedural Pain Management 2nd Edition. *Paediatric Anaesthesia*, *22*(Supplement 1), 1-79.

Reason for exclusion: Ineligible concept.

102. Dawes, T. R., Eden-Green, B., Rosten, C., Giles, J., Governo, R., Marcelline, F., & Nduka, C. (2018). Objectively measuring pain using facial expression: is the technology finally ready?. *Pain management*, *8*(2), 105-113.

Reason for exclusion: Ineligible concept.

103. Ghazi, A., Far, K. I., Asl, M. E., & Vakili, A. (2018). Comparison of the Effect of Sublingual Buprenorphine and Intravenous Fentanyl on Pain Control after Cesarean Surgery. *Asian Research Journal of Gynaecology and Obstetrics*, 1-10.

Reason for exclusion: Ineligible concept.

104. Levent, S., Soydinc, M. H., Elzem, S., Omer, C., & Mehrican, S. (2017). Comparison of 3 different regional block techniques in pediatric patients. *Saudi Medical Journal*, *38*(9), 952-959.

Reason for exclusion: Ineligible concept.

105. Jones, E. M., Barinsky, G. L., & Johnson, A. P. (2021). Non-Enteral Pain Management. In *Perioperative Pain Control: Tools for Surgeons* (pp. 95-116). Springer, Cham.

Reason for exclusion: Ineligible concept.

106. Ojo, A. (2019). *The Use of Aromatherapy to Improve Post-Operative Perception of Pain* (Doctoral dissertation, Grand Canyon University).

Reason for exclusion: Ineligible concept.

107. Martin, S. S. (2016). *Associations between weight status and post-tonsillectomy pain experiences in children: A retrospective study* (Doctoral dissertation).

Reason for exclusion: Ineligible concept.

108. Altchek, A. (2009). Congenital Anomalies II–"No Eponym Syndrome," and Diagnostic Problems. *Pediatric, Adolescent and Young Adult Gynecology*, 231.

Reason for exclusion: Ineligible concept.

109. Abbasi, M. M., & Kashiyarndi, S. 2006). Clinical Decision Support Systems: A discussion on different methodologies used in Health Care. *Marlaedalen University Sweden*.

Reason for exclusion: Ineligible concept.

110. CRELLIN, D., SANTAMARIA, N., BABL, F. E., & HARRISON, D. Appendix B Randomised controlled trials: Study details.

Reason for exclusion: Ineligible concept.

111. Renfrow, T. A. (2009). *Pediatric intensive care unit pain management in extubated postoperative infants and children*. Northern Kentucky University.

Reason for exclusion: Ineligible concept.

112. Carter, B., & Jonas, D. (2009). Nursing Care and Management of Children's Perioperative Pain. In *Perioperative Care of the Child: A Nursing Manual* (pp. 39-65). John Wiley and Sons.

Reason for exclusion: Ineligible concept.

113. Khanzadeh Alishahi, M. (2018). *Evaluation of intraperitoneal ropivacaine for postoperative analgesia following ovariohysterectomy in dogs* (Doctoral dissertation, University of Pretoria).

Reason for exclusion: Ineligible concept.

114. Katz, J. (2003). Timing of treatment and pre-emptive analgesia. CRC Press.

Reason for exclusion: Ineligible concept.

115. Akural, E. I., Bandschapp, O., Bartels, S., Benson, M., Brown, E. S., Candiotti, K. A., ... & Dashti, G. A. Acetaminophen group.

Reason for exclusion: Ineligible concept.

116. Long, N. H. (2010). Factors related to postoperative symptoms among patients undergoing abdominal surgery. *Master's thesis. Faculty of Nursing, Graduate School, Burapha University, Thailand*.

Reason for exclusion: Ineligible concept.

117. Standing, J. F., Savage, I., Pritchard, D., & Waddington, M. (2015). WITHDRAWN: Diclofenac for acute pain in children. *The Cochrane database of systematic reviews*, (7), CD005538-CD005538.

Reason for exclusion: Ineligible concept.

118. Pitcher, K. (2016). The development of an online resource manual for parents caring for their child at home following surgery.

Reason for exclusion: Ineligible concept.

119. Aida, S., & Shimoji, K. (2000). Pre-emptive analgesia: recent findings. *Pain Reviews*, *7*(2), 105.

Reason for exclusion: Ineligible concept.

120. TEC, A. 7. NONPHARMACOLOGICAL TECHNIQUES.

Reason for exclusion: Ineligible concept.

121. Machoki, M. S. (2015). *Local anesthetic wound infusion versus standard analgesia in paediatric post-operative pain control: a randomised control trial* (Master's thesis, University of Cape Town).

Reason for exclusion: Ineligible concept.

122. Bilal, r. M., durrani, b. H. K., & khan, r. A. S. (2021). Comparison of postoperative pain score following transversus abdominis plane block versus placebo, in patients undergoing elective total abdominal hysterectomy under general anaesthesia. *Pafmj*, *71*(1), 40-44.

Reason for exclusion: Ineligible concept.

123. DOSS, L. H., GOUDA, N. M., NASSAR, H. M., MOHAMED, M. E. A., & HAMZA, H. M. Surgical transversus abdominis plane (TAP) block effect on pain scales in children undergoing splenectomy.

Reason for exclusion: Ineligible concept.

124. Martin, S. D., & John, L. D. (2020). Implications of a Retrospective Study on Weight-Based Risk for Post-Tonsillectomy Pain in Children. *Journal of PeriAnesthesia Nursing*, *35*(2), 140-146.

Reason for exclusion: Ineligible concept.

125. Wilson, L., & Tisdall, C. (2020). The development and implementation of a paediatric pain protocol within the recovery room environment. *Australian Nursing and Midwifery Journal*, *26*(9), 14-17.

Reason for exclusion: Ineligible concept.

126. Shipton, E. A. (2016). The Transition of Acute Postoperative Pain to Acute Persistent Pain to Chronic Pain: Assessing and Managing the Risks. In *Perioperative Medicine–Current Controversies* (pp. 421-452). Springer, Cham.

Reason for exclusion: Ineligible concept.

127. Shiny Priyadarshini, A. (2017). *A Prospective Randomised Control Double blinded study comparing ultrasound guided transversus abdominis plane block vs ultrasound guided ilioinguinal block for postoperative analgesia in children undergoing elective hernia repair surgery* (Doctoral dissertation, Madras Medical College, Chennai).

Reason for exclusion: Ineligible concept.

128. Rokni, A., Eslahtalab, A., Ahmadi, R., Sezavar, M., Gharaei Jomehei, M., Heydari, O., ... & Asadi, N. (2020). The Efficacy of Meperidine for Pain Management in Orthopedic, Dental, and General Surgery in Children: A Review Study. *International Journal of Pediatrics*, *8*(10), 12305-12316.

Reason for exclusion: Ineligible concept.

129. Kossowsky, J., Donado, C., & Berde, C. B. (2015). Immediate rescue designs in pediatric analgesic trials: a systematic review and meta-analysis. *Anesthesiology*, *122*(1), 150-171.

Reason for exclusion: Ineligible concept.

130. Kless, J. R. (2010). *Factors associated with moderate and severe postoperative pain* (Doctoral dissertation, Case Western Reserve University).

Reason for exclusion: Ineligible concept.

131. Tobias, J. D. (2000). Weak analgesics and nonsteroidal anti-inflammatory agents in the management of children with acute pain. *Pediatric Clinics of North America*, *47*(3), 527-543.

Reason for exclusion: Ineligible concept.

132. Zurawin, R. K. (2009). Laparoscopy in the Pediatric and Adolescent Female. *Pediatric, Adolescent and Young Adult Gynecology*, 246.

Reason for exclusion: Ineligible concept.

133. Wise, S. S. (2008). Management of Perioperative Pain. In *Surgery* (pp. 381-393). Springer, New York, NY.

Reason for exclusion: Ineligible concept.

134. Khalil, N., & Marouf, H. M. (2017). Paravertebral Block vs. Caudal Block Using Dexmedetomidine Plus Local Anesthetics for Inguinal Hernia Repair in Pediatrics: A Randomized Prospective Trial. *J Anesth Clin Res*, *8*(748), 2.

Reason for exclusion: Ineligible concept.

135. Ceelie, I. (2011). Pain: Postoperative Analgesia in Infants and Neonates.

Reason for exclusion: Ineligible concept.

136. Dehghan, K., Ghalizadeh, M., Macooie, A., & Foroutan, H. (2019). The Comparison of the Analgesic Effect of Intravenous Acetaminophen with Fentanyl in Thoracic and Abdominal Surgeries of Newborns. *International Journal of Pediatrics*, *7*(7), 9773-9781.

Reason for exclusion: Ineligible concept.

137. Oliver, L. A., Oliver, J. A., Rayaz, H., & Lichtor, J. L. (2018). Pediatric Pain. In *Essentials of Regional Anesthesia* (pp. 655-682). Springer, Cham.

Reason for exclusion: Ineligible concept.

138. Blakely, M. L., Kao, L. S., Tsao, K., Huang, E. Y., Tsai, A., Tanaka, S., ... & Collaborative, P. S. R. (2013). Adherence of randomized trials within children's surgical specialties published during 2000 to 2009 to standard reporting guidelines. *Journal of the American College of Surgeons*, *217*(3), 394-399.

Reason for exclusion: Ineligible concept.

139. Voepel-Lewis, T., Zikmund-Fisher, B. J., Boyd, C. J., Veliz, P. T., McCabe, S. E., Weber, M., & Tait, A. R. (2018). Effect of a scenario-tailored opioid messaging program on parents' risk perceptions and opioid decision-making. *The Clinical journal of pain*, *34*(6), 497.

Reason for exclusion: Ineligible concept.

140. Johnson, M. I. (2017). Transcutaneous electrical nerve stimulation (TENS) as an adjunct for pain management in perioperative settings: a critical review. *Expert review of neurotherapeutics*, *17*(10), 1013-1027.

Reason for exclusion: Ineligible concept.

141. O'Hara, D. A., Fanciullo, G., Hubbard, L., Maneatis, T., Seuffert, P., Bynum, L., & Shefrin, A. (1997). Evaluation of the safety and efficacy of ketorolac versus morphine by patient-controlled analgesia for postoperative pain. *Pharmacotherapy: The Journal of Human Pharmacology and Drug Therapy*, *17*(5), 891-899.

Reason for exclusion: Ineligible concept.

142. Shipton, E. A. (2014). The transition of acute postoperative pain to chronic pain: Part 1–Risk factors for the development of postoperative acute persistent pain. *Trends in Anaesthesia and Critical Care*, *4*(2-3), 67-70.

Reason for exclusion: Ineligible concept.

143. Mittal, S., Shukla, A. R., Sahadev, R., Lee, S. Y., Siu, S., Gale, E. M., ... & Srinivasan, A. K. (2020). Reducing post-operative opioids in children undergoing outpatient urologic surgery: A quality improvement initiative. *Journal of Pediatric Urology*, *16*(6), 846-e1.

Reason for exclusion: Ineligible concept.

144. Lacher, M., Kuebler, J. F., Dingemann, J., & Ure, B. M. (2014, October). Minimal invasive surgery in the newborn: current status and evidence. In *Seminars in pediatric surgery* (Vol. 23, No. 5, pp. 249-256). WB Saunders.

Reason for exclusion: Ineligible concept.

145. Campbell, F. (2014). Improving postoperative pain outcomes for children. In *International Forum on Pediatric Pain*.

Reason for exclusion: Ineligible concept.

146. Aubrey, t. Y., janette, t., & michael salvador, d. Comparison of postoperative pain between ultrasound-guided quadratus lumborum block versus ultrasound-guided caudal epidural block in children 1-6 years old undergoing unilateral lower abdominal and urological surgeries in philippine children's medical center: a randomized controlled trial. *An official publication of the philippine children's medical center*, *16*(1).

Reason for exclusion: Ineligible concept.

147. Merella, F., Canchi-Murali, N., & Mossetti, V. (2019). General principles of regional anaesthesia in children. *Bja Education*, *19*(10), 342-348.

Reason for exclusion: Ineligible concept.

148. Yang, K., Baetzel, A., Chimbira, W. T., Yermolina, Y., Reynolds, P. I., & Nafiu, O. O. (2017). Association of sleep disordered breathing symptoms with early postoperative analgesic requirement in pediatric ambulatory surgical patients. *International journal of pediatric otorhinolaryngology*, *96*, 145-151.

Reason for exclusion: Ineligible concept.

149. Aun, C. S., & Karmakar, M. K. (2008). Anesthesia for Pediatric Laparoscopy. In *Endoscopic Surgery in Infants and Children* (pp. 229-232). Springer, Berlin, Heidelberg.

Reason for exclusion: Ineligible concept.

150. Cravero, J. P., Agarwal, R., Berde, C., Birmingham, P., Coté, C. J., Galinkin, J., ... & Wilder, R. (2019). The Society for Pediatric Anesthesia recommendations for the use of opioids in children during the perioperative period. *Pediatric Anesthesia*, *29*(6), 547-571.

Reason for exclusion: Ineligible concept.

151. Sahin, L., Soydinc, M. H., Sen, E., Cavus, O., & Sahin, M. (2017). Comparison of 3 different regional block techniques in pediatric patients: A prospective randomized single-blinded study. *Saudi medical journal*, *38*(9), 952.

Reason for exclusion: Ineligible concept.

152. Simanski, C. J., Althaus, A., Hoederath, S., Kreutz, K. W., Hoederath, P., Lefering, R., ... & Neugebauer, E. A. (2014). Incidence of chronic postsurgical pain (CPSP) after general surgery. *Pain medicine*, *15*(7), 1222-1229.

Reason for exclusion: Ineligible concept.

153. Prot-Labarthe, S., Pelletier, É., Winterfeld, U., Villeneuve, E., Wood, C., Bussières, J. F., ... & Bourdon, O. (2008). Comparison of pain management in paediatric surgical patients in two hospitals in France and Canada. *Pharmacy World & Science*, *30*(3), 251-257.

Reason for exclusion: Ineligible concept.

154. Ingelmo, P., Rivera, G., & Baird, R. (2016). Pain control after pediatric surgery: learning from the past to perfect the future.

Reason for exclusion: Ineligible concept.

155. Hossain, T., & Huq, M. A. U. (2013). Pediatric Laparoscopic Surgery: Four Years' Experience in Dhaka Medical College Hospital. *Journal of Paediatric Surgeons of Bangladesh*, *4*(1), 11-18.

Reason for exclusion: Ineligible concept.

156. Sophia, P., Prasad, P. K., Kumar, S. K., & Lakshmi, B. S. (2014). To evaluate the analgesic efficacy of ipsilateral transversus abdominis plane block for lower abdominal surgeries in children: a prospective randomised controlled study. *Journal of Evolution of Medical and Dental Sciences*, *3*(28), 7682-7692.

Reason for exclusion: Ineligible concept.

157. Shokri, H. (2017). The efficacy of rectus sheath block for pain management following laparoscopic orchiopexy surgery. *Ain-Shams Journal of Anaesthesiology*, *10*(1), 219.

Reason for exclusion: Ineligible concept.

158. Kolarkar, P., Badwaik, G., Watve, A., Agarkar, S., Kalbande, J., Bhangale, N., & Bhalerao, A. (2014). Pre-emptive oral clonidine for immediate postoperative pain in surgeries under sub-arachnoid block. *Journal of Evolution of Medical and Dental Sciences*, *3*(51), 11913-11922.

Reason for exclusion: Ineligible concept.

159. Deer, J. D., Sawardekar, A., & Suresh, S. (2016). Day surgery regional anesthesia in children: safety and improving outcomes, do they make a difference?. *Current Opinion in Anesthesiology*, *29*(6), 691-695.

Reason for exclusion: Ineligible concept.

160. Nascimento, L. C., Warnock, F., Pan, R., Silva-Rodrigues, F. M., Castral, T. C., De Bortoli, P. S., ... & Scochi, C. G. S. (2019). Parents' participation in managing their children's postoperative pain at home: an integrative literature review. *Pain Management Nursing*, *20*(5), 444-454.

Reason for exclusion: Ineligible concept.

161. Mostafa, M. F., Hamed, E., Amin, A. H., & Herdan, R. (2020). Dexmedetomidine versus clonidine adjuvants to levobupivacaine for ultrasound-guided transversus abdominis plane block in paediatric laparoscopic orchiopexy: Randomized, double-blind study. *European Journal of Pain*.

Reason for exclusion: Ineligible concept.

162. Bryskin, R. B., Londergan, B., Wheatley, R., Heng, R., Lewis, M., Barraza, M., ... & Ye, G. (2015). Transversus abdominis plane block versus caudal epidural for lower abdominal surgery in children: a double-blinded randomized controlled trial. *Anesthesia & Analgesia*, *121*(2), 471-478.

Reason for exclusion: Ineligible concept.

163. Schultz-Machata, A. M., Weiss, M., & Becke, K. (2014). What's new in pediatric acute pain therapy?. *Current Opinion in Anesthesiology*, *27*(3), 316-322.

Reason for exclusion: Ineligible concept.

164. Russell, P., von9 Ungern-Sternberg, B. S., & Schug, S. A. (2013). Perioperative analgesia in pediatric surgery. *Current Opinion in Anesthesiology*, *26*(4), 420-427.

Reason for exclusion: Ineligible concept.

165. Anwer, Z. M. (2020). Comparing the Efficacy of Paracetamol, Diclofenac, and Ketorolac on Post-Appendectomy Outcomes in Children and Adolescents. *Iraqi Journal of Pharmaceutical Sciences (P-ISSN: 1683-3597, E-ISSN: 2521-3512)*, *29*(1), 123-133.

Reason for exclusion: Duplicate (similar information).

166. Hossain, T., & Huq, A. U. (2013). Pediatric laparoscopic surgery: three years experience in a tertiary level hospital in Dhaka, Bangladesh. *Bangladesh Journal of Endosurgery*, *1*(1), 21-25.

Reason for exclusion: Ineligible concept.

167. Kokki, H., Lintula, H., Vanamo, K., Heiskanen, M., & Eskelinen, M. (2005). Oxycodone vs placebo in children with undifferentiated abdominal pain: a randomized, double-blind clinical trial of the effect of analgesia on diagnostic accuracy. *Archives of pediatrics & adolescent medicine*, *159*(4), 320-325.

Reason for exclusion: Ineligible concept.

168. Manworren, R. C. (2015). Multimodal pain management and the future of a personalized medicine approach to pain. *Aorn Journal*, *101*(3), 307-318.

Reason for exclusion: Ineligible concept.

Kristin, N., Schönfeld, C. L., Bechmann, M., Bengisu, M., Ludwig, K., Scheider, A., & Kampik, A. (2001). Vitreoretinal surgery: pre-emptive analgesia. *British journal of ophthalmology*, *85*(11), 1328-1331.

Reason for exclusion: Ineligible concept.

169. Bazin, V., Bollot, J., Asehnoune, K., Roquilly, A., Guillaud, C., De Windt, A., ... & Lejus, C. (2010). Effects of perioperative intravenous low dose of ketamine on postoperative analgesia in children. *European Journal of Anaesthesiology| EJA*, *27*(1), 47-52.

Reason for exclusion: Ineligible concept.

170. Hamill, J. K. *Sprayed Intraperitoneal Local Anesthetic for Laparoscopic Appendectomy in Children* (Doctoral dissertation, School of Medicine, Faculty of Medical and Health Sciences, University of Auckland, Auckland, New Zealand).

Reason for exclusion: Duplicate (similar information).

171. Benito, J., Monteiro, B. P., Beaudry, F., Lavoie, A. M., Lascelles, B. D. X., & Steagall, P. V. (2016). Pharmacokinetics of bupivacaine after intraperitoneal administration to cats undergoing ovariohysterectomy. *American journal of veterinary research*, *77*(6), 641-645.

Reason for exclusion: Ineligible concept.

172. Tian, Y., Wu, X. Y., Li, L., Ma, L., & Li, Y. F. (2017). A clinical trial evaluating the laryngeal mask airway-Supreme in obese children during general anesthesia. *Archives of medical science: AMS*, *13*(1), 183.

Reason for exclusion: Ineligible concept.

173. Bahar, M. M., Jangjoo, A., Soltani, E., Armand, M., & Mozaffari, S. (2010). Effect of preoperative rectal indomethacin on postoperative pain reduction after open cholecystectomy. *Journal of PeriAnesthesia Nursing*, *25*(1), 7-10.

Reason for exclusion: Ineligible concept.

174. Astuto, M., Rosano, G., Rizzo, G., Disma, N., & Di Cataldo, A. (2007). Methodologies for the treatment of acute and chronic nononcologic pain in children. *Minerva anestesiologica*, *73*(9), 459-465.

Reason for exclusion: Ineligible concept.

175. Jitpakdee, T., & Mandee, S. (2014). Strategies for preventing side effects of systemic opioid in postoperative pediatric patients. *Pediatric Anesthesia*, *24*(6), 561-568.

Reason for exclusion: Ineligible concept.

176. Uchinami, Y., Sakuraya, F., Tanaka, N., Hoshino, K., Mikami, E., Ishikawa, T., ... & Morimoto, Y. (2017). Comparison of the analgesic efficacy of ultrasound-guided rectus sheath block and local anesthetic infiltration for laparoscopic percutaneous extraperitoneal closure in children. *Pediatric Anesthesia*, *27*(5), 516-523.

Reason for exclusion: Ineligible concept.

177. Suresh, S., Schaldenbrand, K., Wallis, B., & De Oliveira Jr, G. S. (2014). Regional anaesthesia to improve pain outcomes in paediatric surgical patients: a qualitative systematic review of randomized controlled trials. *British journal of anaesthesia*, *113*(3), 375-390.

Reason for exclusion: Ineligible concept.

178. Moyao-García, D., Hernández-Palacios, J. C., Ramírez-Mora, J. C., & Nava-Ocampo, A. A. (2009). A pilot study of nalbuphine versus tramadol administered through continuous intravenous infusion for postoperative pain control in children. *Acta Biomed*, *80*(80), 124-130.

Reason for exclusion: Ineligible concept.

179. Mahdi, E. M., Ourshalimian, S., Russell, C. J., Zamora, A. K., & Kelley-Quon, L. I. (2020). Fewer postoperative opioids are associated with decreased duration of stay for children with perforated appendicitis. *Surgery*, *168*(5), 942-947.

Reason for exclusion: Ineligible concept.

180. Batley, S. E., Prasad, V., Vasdev, N., & Mohan-S, G. (2015). Post-operative pain management in patients undergoing robotic urological surgery. *Current urology*, *9*(1), 5-11.

Reason for exclusion: Ineligible concept.

181. Carney, D. E., Nicolette, L. A., Ratner, M. H., Minerd, A., & Baesl, T. J. (2001). Ketorolac reduces postoperative narcotic requirements. *Journal of pediatric surgery*, *36*(1), 76-79.

Reason for exclusion: Ineligible concept.

182. El Fawy, D. M., & El Gendy, H. A. (2014). Ultrasound-guided transversus abdominis plane block versus caudal block for postoperative pain relief in infants and children undergoing surgical pyeloplasty. *Ain-Shams Journal of Anaesthesiology*, *7*(2), 177.

Reason for exclusion: Ineligible concept.

183. Kendall, M. C., Alves, L. J. C., Suh, E. I., McCormick, Z. L., & De Oliveira, G. S. (2018). Regional anesthesia to ameliorate postoperative analgesia outcomes in pediatric surgical patients: an updated systematic review of randomized controlled trials. *Local and regional anesthesia*, *11*, 91.

Reason for exclusion: Ineligible concept.

184. Dingeman, R. S., Barus, L. M., Chung, H. K., Clendenin, D. J., Lee, C. S., Tracy, S., ... & Chen, C. (20113). Ultrasonography-guided bilateral rectus sheath block vs local anesthetic infiltration after pediatric umbilical hernia repair: a prospective randomized clinical trial. *JAMA surgery*, *148*(8), 707-713.

Reason for exclusion: Ineligible concept.

185. Crellin, D. J., Harrison, D., Santamaria, N., & Babl, F. E. (2015). Systematic review of the Face, Legs, Activity, Cry and Consolability scale for assessing pain in infants and children: is it reliable, valid, and feasible for use?. *Pain*, *156*(11), 2132-2151.

Reason for exclusion: Ineligible type of study (Systematic review)

186. Pennant, J. H. (2001). Anesthesia for laparoscopy in the pediatric patient. *Anesthesiology Clinics of North America*, *19*(1), 69-88.

Reason for exclusion: Ineligible concept.

187. 1Billings, K. R., Manworren, R. C., Lavin, J., Stake, C., Hebal, F., Leon, A. H., & Barsness, K. (2019). Pediatric emergency department visits for uncontrolled pain in postoperative adenotonsillectomy patients. *Laryngoscope investigative otolaryngology*, *4*(1), 165-169.

Reason for exclusion: Ineligible concept.

188. Batko, I., Kościelniak-Merak, B., Al-Mutari, I., & Kobylarz, K. (2017). Benefits of ultrasound-guided transversus abdominis plane block for open appendectomy in children. *Anaesthesiology intensive therapy*, *49*(3).

Reason for exclusion: Ineligible concept.

189. El Basha, S., Hanna, M. G., Soaida, S. M., & Refaee, H. H. (2015). Intraperitoneal instillation of l-bupivacaine in laparoscopic pediatric procedures: a randomized-controlled study. *Ain-Shams Journal of Anaesthesiology*, *8*(2), 247.

Reason for exclusion: Ineligible concept.

190. Zhu, A., Benzon, H. A., & Anderson, T. A. (2017). Evidence for the efficacy of systemic opioid-sparing analgesics in pediatric surgical populations: a systematic review. *Anesthesia & Analgesia*, *125*(5), 1569-1587.

Reason for exclusion: Ineligible type of study (Systematic review)

191. Fortier, M. A., Chou, J., Maurer, E. L., & Kain, Z. N. (2011). Acute to chronic postoperative pain in children: preliminary findings. *Journal of pediatric surgery*, *46*(9), 1700-1705.

Reason for exclusion: Ineligible concept.

192. Reismann, M., von Kampen, M., Laupichler, B., Suempelmann, R., Schmidt, A. I., & Ure, B. M. (2007). Fast-track surgery in infants and children. *Journal of pediatric surgery*, *42*(1), 234-238.

Reason for exclusion: Ineligible concept.

193. Kokki, H. (2003). Nonsteroidal anti-inflammatory drugs for postoperative pain. *Pediatric Drugs*, *5*(2), 103-123.

Reason for exclusion: Ineligible concept.

194. Di Pace, M. R., Cimador, M., Catalano, P., Caruso, A., Sergio, M., Casuccio, A., & De Grazia, E. (2009). Efficacy of periportal infiltration and intraperitoneal instillation of ropivacaine after laparoscopic surgery in children. *Journal of Laparoendoscopic & Advanced Surgical Techniques*, *19*(6), 821-825.

Reason for exclusion: Ineligible concept.

195. McHoney, M., Wade, A. M., Eaton, S., Howard, R. F., Kiely, E. M., Drake, D. P., ... & Pierro, A. (2011). Clinical outcome of a randomized controlled blinded trial of open versus laparoscopic Nissen fundoplication in infants and children. *Annals of surgery*, *254*(2), 209-216.

Reason for exclusion: Ineligible concept.

196. Palabiyik, O., & Demir, G. (2021). Chronic Pain after Open Appendectomy and Its Effects on Quality of Life in Children Aged 8–18 Years. *Pain Research and Management*, *2021*.

Reason for exclusion: Ineligible concept.

197. Ong, C. K. S., Lirk, P., Seymour, R. A., & Jenkins, B. J. (2005). The efficacy of preemptive analgesia for acute postoperative pain management: a meta-analysis. *Anesthesia & Analgesia*, *100*(3), 757-773.

Reason for exclusion: Ineligible concept.

198. Riad, W., & Moussa, A. (2007). Pre-operative analgesia with rectal diclofenac and/or paracetamol in children undergoing inguinal hernia repair. *Anaesthesia*, *62*(12), 1241-1245.

Reason for exclusion: Ineligible concept.

199. Choi, G. J., Kang, H., Kim, B. G., Choi, Y. S., Kim, J. Y., & Lee, S. (2017). Pain after single-incision versus conventional laparoscopic appendectomy: a propensity-matched analysis. *journal of surgical research*, *212*, 122-129.

Reason for exclusion: Ineligible concept.

Supplementary File S3: Characteristics of the included studies

Authors	Year	Country	Purpose	Participants	Study method	POP
Liu et al	2018 - 2019	China	Effect of LA & LP on recovery, inflammation, and oxidative stress	115	Clinical	§
Kaszynski et al	2019 - 2020	Poland	Lidocaine infusions for pain relief undergoing LA	71	RCT	¥
Alkhoury et al	2010 - 2011	USA	Discharge effect on patient and parent satisfaction	207	Prospective	§
Applegate et al	1998 - 2012	USA	Acute vs interval surgery in terms of POP	180	Retrospective	§
Majeed et al	2018 - 2019	Iraq	Comparison of analgesics after LA	120	Clinical	§
Sandeman et al	2008 - 2009	Australia	UGTAP-block for LA	93	PRT	€
Maloney et al	2014 – 2015	USA	UGBRSB vs local analgesia	275	Retrospective	§
Till et al	NA	Germany	PCA for POP management	90	Prospective	§
Tomecka et al	2004 - 2010	USA	POP after LA	186	Retrospective	NA
Norton et al	NA	USA	OA vs LA	223	Retrospective	§
Liu et al	2011 - 2012	USA	MMAT after LA for POP management	206	Retrospective	§
Wilson et al	NA	Australia	Management of POP after LA/OA	76	Prospective	€
Sola et al	NA	USA	IV-Acetaminophen for POP after LA	82	PRT	€
Perez et al	2009 - 2011	USA	SILS vs LA	50	PRT	€
Elnabtity et al	2016 - 2017	Egypt	Intraperitoneal dexmedetomidine as an adjuvant to bupivacaine for POP	52	PRT	§
Hamill et al	NA	New Zealand	Sprayed bupivacaine vs control for POP	175	RCT	€
Ellatif et al	2018 - 2019	Egypt	UGQLB vs TAP block	34	PRT	§
Hu et al	NA	China	Effect of dezocine on POP after LA	60	RCT	§

POP (Postoperative pain), **LA** (Laparoscopic appendectomy), **LP** (Laparotomy), **§** (Significant), **¥** (Little difference), **€** (No difference), **PRT** (Prospective randomized trial), **RCT** (Randomized control trial), **PCA** (Patient controlled analgesia), **OA** (Open appendectomy), **LAI** (Laparoscopic appendectomy infiltration), **MMAT** (Multimodal analgesic treatment), **SILS** (Single-incision laparoscopic surgery), **UGQLB** (Ultrasound-guided quadratus lumborum block), **UGTAP** (Ultrasound-guided transverse abdominis plane).
